# DefensePredictor: A Machine Learning Model to Discover Prokaryotic Immune Systems

**DOI:** 10.1126/science.adv7924

**Published:** 2026-04-02

**Authors:** Peter C. DeWeirdt, Emily M. Mahoney, Michael T. Laub

**Affiliations:** 1 Department of Biology, Massachusetts Institute of Technology; Cambridge, MA 02139, USA.; 2 Computational and Systems Biology Program, Massachusetts Institute of Technology; Cambridge, MA 02139, USA.; 3 Molecular Biology and Genetics Department, Johns Hopkins School of Medicine; Baltimore, MD 21205,USA.; 4 Howard Hughes Medical Institute, Massachusetts Institute of Technology; Cambridge, MA 02139, USA.

## Abstract

Anti-phage defense systems protect bacteria from viral infection and have inspired important biotechnologies such as CRISPR-Cas9 while also revealing the evolutionary roots of eukaryotic innate immunity. Many systems have been discovered by genomic co-localization, but this approach cannot identify systems outside defense islands. We present DefensePredictor, a machine-learning model that uses protein language model embeddings to classify proteins as defensive. Applying DefensePredictor to 69 diverse *E. coli* strains, we predict hundreds of previously unknown systems and experimentally validate 42. Analysis of 1,000 diverse prokaryotic genomes identifies nearly 3,000 protein clusters lacking homology to known systems, revealing a vast, uncharacterized defense repertoire. DefensePredictor will facilitate comprehensive discovery of anti-phage defense systems, which promises to reveal additional connections between prokaryotic and eukaryotic immunity, and accelerate biotechnology development.

Bacteria face a relentless battle with viruses known as bacteriophages. In some environments, these phages can drive the turnover of 10–25% of all bacteria on a daily basis (1). The intense selective pressure to evade or survive infection has driven the evolution of numerous anti-phage defense systems, including restriction-modification and CRISPR-Cas systems. Owing to their molecular specificity, these systems have been powerfully repurposed for genetic and genome engineering. Identifying new anti-phage defense systems may yield the next generation of precision molecular tools, while also shedding important light on the ongoing arms race between bacteria and phages. Notably, recent work has also indicated that many components of the mammalian innate immune system are homologous to, and likely originate from, bacterial proteins that function in anti-phage defense (2, 3).

The full complement of bacterial anti-phage defense systems remains unknown. Several systematic searches for new defense systems leveraged the tendency of some defense genes to co-localize in genomes by looking for uncharacterized genes in these so-called ‘defense islands’ (4). These guilt-by-association methods have identified and validated 59 defense systems (5–7). A complementary experimental approach involving the screening of a library of genomic fragments from diverse *Escherichia coli* genomes identified 21 new defense systems, most of which reside within mobile genetic elements rather than defense islands (8). Other studies have bioinformatically mined mobile elements to identify yet more systems (9–12).

Despite their successes, these prior approaches have fundamental limitations. The guilt-by-association approach focuses exclusively on systems near known defense genes, and not all systems are located in defense islands (8). The experimental screening approach is laborious and not done to saturation. Thus, the full repertoire of anti-phage defense systems in bacteria remains unknown, and we currently lack the tools to systematically identify systems with high speed, sensitivity, and specificity.

Here, we built a machine learning model called DefensePredictor that can predict defense systems. Our model is a gradient boosting classifier built on top of embeddings from a protein language model (PLM). PLMs learn statistical relationships between amino acids within a protein by predicting the identity of masked amino acids from the surrounding protein context (13). DefensePredictor outperforms a guilt-by-association approach at predicting held-out defense genes. We used our model to identify defense genes in diverse strains of *E. coli* with an experimental validation rate of 45%, yielding 42 previously unknown defense systems. Some of the identified systems feature domains, *e.g*. nucleases, found in previous defense systems but in different contexts or configurations. Seven systems contain domains not previously implicated in defense, and we show each is essential for protection. Collectively, our findings demonstrate that DefensePredictor represents a robust and powerful pipeline for identifying anti-phage defense systems. We provide DefensePredictor as an open-source Python package, enabling the rapid prediction of defense systems in any prokaryotic genome in under five minutes.

## DefensePredictor achieves high precision and recall on held-out folds

**Fig. 1.**
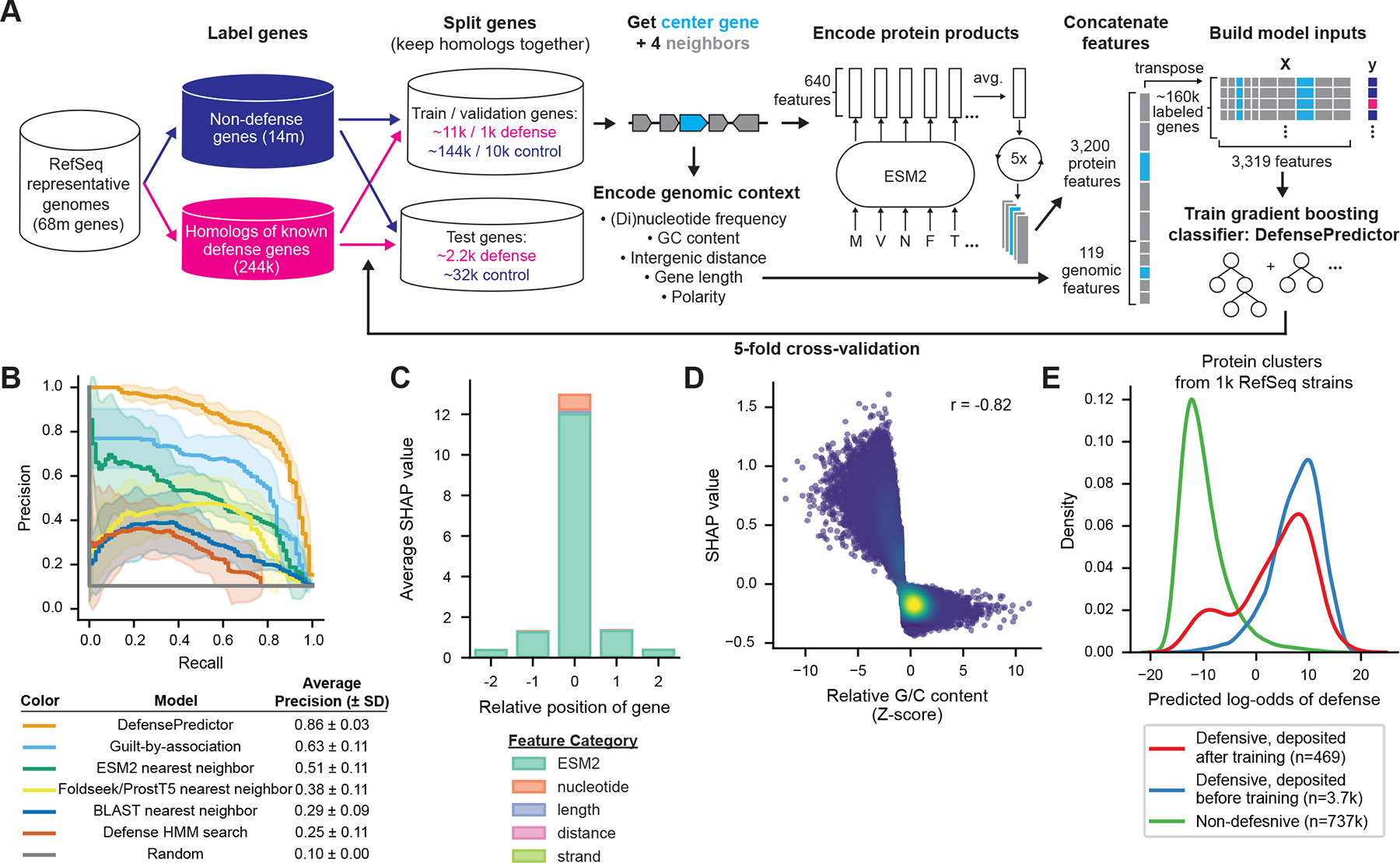
DefensePredictor shows strong performance on held-out folds. (**A**) Pipeline for building DefensePredictor. Train, validation, and test sizes are shown for fold four, which has the median number of defense genes across five folds. Dark blue elements represent control genes, pink elements represent known defense genes. Light blue elements represent center genes, grey elements represent neighboring genes. M, million; k, thousand. (**B**) Precision-recall curves averaged across held-out folds. TP, True Positive; FP, False Positive; FN, False Negative; SD, standard deviation. (**C**) SHAP values summed for each true positive gene (n = 10,307) and feature category and averaged across all true positive genes in the held-out folds. Position 0 represents the center gene; other positions are neighboring genes. (**D**) SHAP value of GC content versus z-scored GC content for all center genes in held-out folds. Color represents density, with yellow being the most dense area of the plot. Pearson correlation is indicated. (**E**) Density plot of DefensePredictor log-odds for defense genes deposited before and after dataset construction and non-defense genes. The top prediction for each protein cluster across 1,000 randomly selected Refseq genomes was used to generate densities. Densities are not scaled between groups. The number of genes in each category is indicated.

To build a machine learning model to predict defense genes, we first searched ~17,000 assembled genomes, representative of the taxonomic diversity of prokaryotes, for known defense systems using DefenseFinder (10) (Fig. 1A). We identified ~244,000 homologs that belong to complete defense systems. These genes represented our positive set for machine learning. To obtain our negative gene set, we identified ~14 million genes with gene ontology (GO) or clusters of orthologous genes (COG) annotations typically associated with non-defensive functions, *e.g*., translation, membrane transport, etc. To reduce redundancy in both the positive and negative gene sets, we clustered proteins with 30% sequence identity and 80% reciprocal coverage using MMseqs2 clustering (14), and randomly selected one protein per cluster, resulting in a dataset of ~186,000 control proteins and ~15,000 defense proteins.

To assess the model’s ability to predict defense proteins that are not homologs of known ones, we divided our dataset (control and defense proteins) into five folds based on homology. We performed an all-by-all MMseqs2 profile search to cluster proteins more sensitively than the MMseqs2 clustering performed above. We then divided our dataset such that homologs remained together in each fold (see Methods, table S1). To encode genes for machine learning, we took each gene in the dataset (hereafter called the ‘center gene’) and its two neighboring genes on either side. For each center and neighboring gene, we built a representation of its protein product using the PLM Evolutionary Scale Model 2 (ESM2). ESM2 generates a 640-dimensional embedding for each amino acid in a protein, which we averaged across each coding sequence to obtain protein-level representations (13, 15). We concatenated the average embeddings for each center gene and its four gene neighbors to obtain a 3,200-dimensional vector of protein features. To also capture genomic context information, for each center gene and its gene neighbors we calculated nucleotide and dinucleotide frequencies, GC content relative to the genome, gene length, the distance between each pair of consecutive genes, and orientation of the neighboring genes relative to the center gene, yielding 119 genomic features. We concatenated the genomic and protein features to obtain a final feature vector size of 3,319. We calculated these features for all ~200,000 genes in our dataset.

Next, we performed five-fold cross validation with our dataset, holding one fold out at a time as a test set and training a gradient boosting classifier on the remaining four folds (16). Each classifier combines predictions from thousands of decision trees, which partition genes based on their feature values and estimate a probability of defense for each partition. We also created validation sets comprised of a subset of defensive and control proteins from training folds, which we used to tune hyperparameters using a Bayesian optimization framework (17). We refer to the resulting ensemble of five models as DefensePredictor.

To evaluate the performance of DefensePredictor, we assessed each of its five constituent models on their respective held-out test fold. Using the true labels for genes in each held-out fold, we calculated the precision (the proportion of predicted defense genes that are actually defensive) and recall (the proportion of actual defense genes that are predicted) over descending probability thresholds (Fig. 1B). To mimic the discovery setting, we calculated precision-recall curves using only the single highest-scoring gene from each defense HMM (hidden Markov models) cluster and from each non-defense functional group (GO or COG). As a summary statistic, we calculated the average precision (AP) across all recall cutoffs. We found that DefensePredictor achieved a mean AP of 0.86 across all five folds, highlighting its ability to recover unseen defense genes with high precision and recall.

To ensure that there was minimal leakage of homologs between folds, we evaluated several homology-based approaches for identifying held-out defense proteins. In the first approach, we aligned proteins in held-out folds with the HMMs that were used to identify defense proteins in the other four folds. We then ranked held-out proteins by the bit-score of their most significant alignment. This approach achieved a mean AP of 0.25. In the second homology-based approach, we aligned proteins in the held-out folds with all proteins in the other four folds using BLAST. We then ranked held-out proteins based on the bit-score of their most significant alignment, taking the negative bit-score for proteins whose best hit was a non-defensive protein. This approach yielded a mean AP of 0.29. Finally, we evaluated a structure-based homology approach by searching the predicted structural representations of proteins in held-out folds against the structural representations of proteins in the other four folds using Foldseek and ProstT5 (18). We then ranked proteins in the same way as the BLAST approach. This structural search yielded a mean AP of 0.38. Together, these homology-based searches suggest there is minimal leakage between cross-validation folds, and thus, that DefensePredictor can identify defense proteins that are not simply homologs of known defense proteins.

We next compared DefensePredictor to a guilt-by-association method. To rank genes with this method, we returned to the full dataset of ~17,000 genomes and calculated how frequently genes in each cluster (defined above using MMseqs2 clustering) were encoded near known defense genes. We did not count defensive neighbors that belonged to the same system as the query gene. Furthermore, to make the comparison fair with DefensePredictor, we did not count defensive neighbors that were in the same fold as the query gene. We ranked genes based on the significance of their cluster’s association (measured using a one-sided Fisher’s exact test) and calculated the precision and recall at descending significance thresholds. Using this method, we obtained a mean AP of 0.63 across folds, a lower value than with DefensePredictor (AP = 0.86). We also compared this guilt-by-association approach with DefensePredictor using alternative evaluation metrics. We calculated the area under the receiver operating characteristic curve, where DefensePredictor achieved a mean value of 0.97 versus 0.91 for guilt-by-association (table S2). Similarly, DefensePredictor outperformed the guilt-by-association method when evaluated using a top 10 precision metric (1.0 versus 0.78) and a top 60 precision metric (0.83 versus 0.69). These results suggest that DefensePredictor is more powerful for identifying unknown defense genes than guilt-by-association.

Next, we aimed to understand the individual features that DefensePredictor used to identify defense proteins. To do so, we calculated SHapley Additive exPlanation (SHAP) values, which estimate how input features contribute to a model’s predictions (19). Averaging SHAP values across all five folds revealed that ESM2 features of the center gene were the most important contributor to predictions (Fig. 1C; table S3).

Given their importance for making predictions, we wondered whether ESM2 embeddings alone could identify new defense proteins. To test this, we ranked held-out proteins in each fold by the cosine similarity between their ESM2 embedding and the embedding of their nearest neighbor in the other four folds, taking the negative cosine similarity for proteins whose nearest neighbor was non-defensive. This approach yielded a mean AP of 0.51, suggesting that ESM2 embeddings alone capture a substantial portion of the information necessary to identify new defense proteins, confirming their importance from the SHAP value analysis. The information that ESM2 captures likely extends beyond homology, as evidenced by this approach’s superior performance to the homology-based approaches. However, the finding that DefensePredictor outperforms ESM2 cosine similarity indicates that incorporating genomic features, features for neighboring genes, and supervised learning improves predictions.

While it is difficult to pinpoint what biological information the ESM2 features capture, we made several observations that suggest DefensePredictor has learned biologically meaningful patterns to make its predictions. First, the most important non-ESM2 feature (fourth most important overall) is the GC content of the center gene (Fig. 1D). Indeed, it has previously been observed that defense genes tend to have lower GC content than their host genomes, likely reflecting their acquisition by lateral gene transfer (20, 21) (fig. S1A). Further, when we examined which neighboring genes DefensePredictor leveraged to make its predictions, by summing SHAP values for individual neighboring genes and annotating the genes that contribute most positively to predictions (table S3), we noted an enrichment of neighboring genes annotated as anti-phage defensive or encoding transposases or integrases (fig. S1B). These results suggest that DefensePredictor learned to leverage the well-described clustering of defense genes in islands (5–7) and the association between defense genes and mobile elements (4, 9) to make its predictions. Together these observations suggest that DefensePredictor has learned biologically relevant patterns to make its predictions.

To prospectively validate DefensePredictor, we evaluated it against defense genes deposited in DefenseFinder after our training dataset was constructed. We ran predictions on 1,000 randomly selected prokaryotic RefSeq strains (table S4). We then used an updated version of DefenseFinder to identify newly deposited defense systems, excluding systems discovered in this work. This search revealed 1,178 instances of these systems across the 1,000 genomes, each one matching to one of 100 distinct, newly deposited systems. For each of these 100 systems, we considered its top scoring gene across all instances. 82 distinct systems had a maximum probability of defense greater than 0.5 (Fig. 1E), and 68 systems had a maximum probability of defense greater than a more stringent cutoff of 0.98. We will refer to this stringent cutoff by its log-odds value of 4, where the log-odds of a gene is defined as $\text{log}\left(\frac{p}{1-p}\right)$, with *p* being its predicted probability of defense. Across 122 instances of newly deposited systems with multiple genes and at least one predicted defense gene (log-odds > 0), 95 (78%) had all genes in the multi-gene system predicted as defensive. On the other hand, in 27 of these instances at least one gene was not predicted as defensive, suggesting that sometimes new defense genes can be uncovered by searching for genes in the same operon as a predicted defense gene. These results confirm that DefensePredictor can identify new, previously unknown defense systems.

## Prediction of defense genes in a diverse collection of *E. coli* strains

**Fig. 2.**
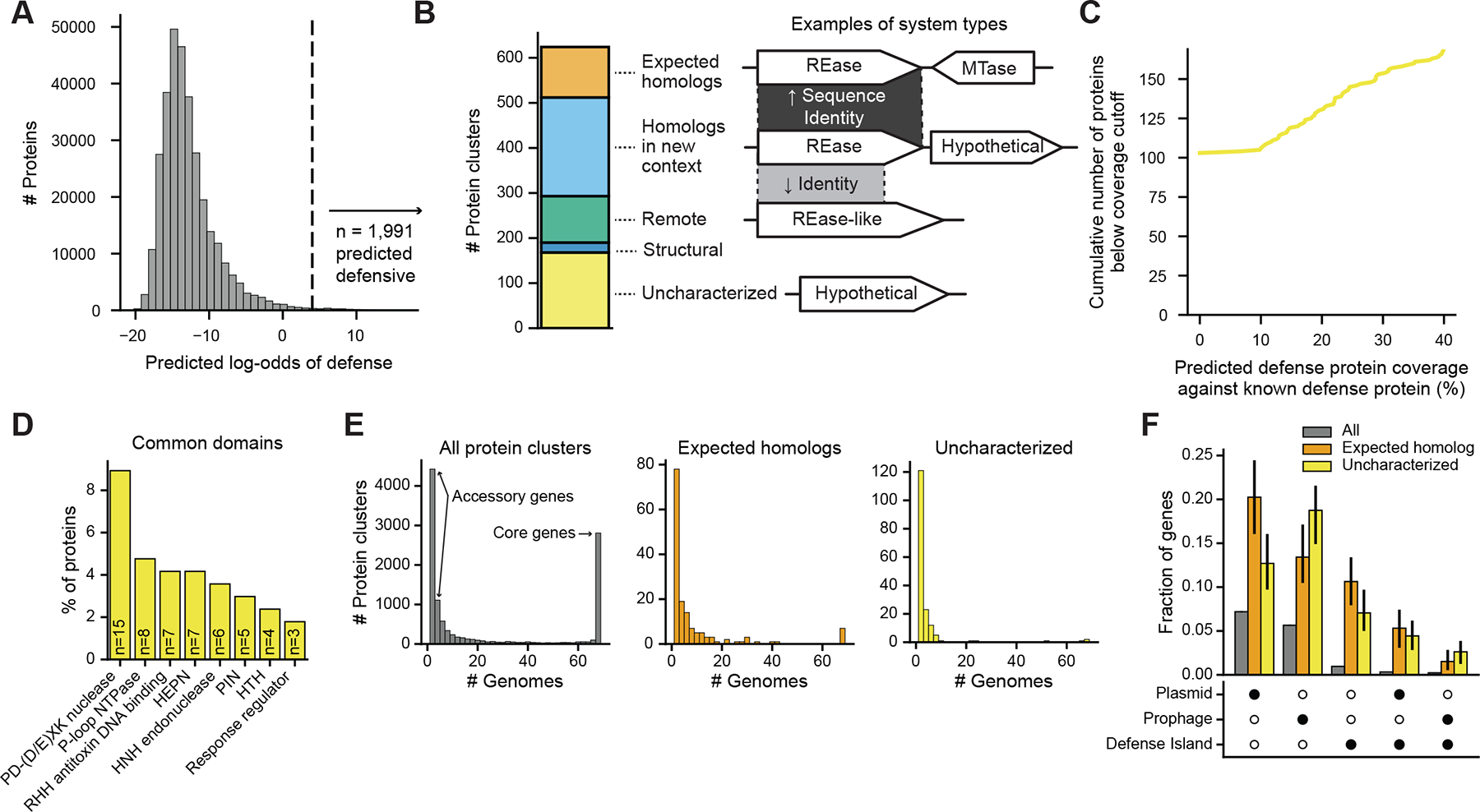
DefensePredictor identifies hundreds of predicted defense genes in 69 diverse *E. coli* strains. (**A**) Histogram of log-odds for 321,347 genes from 69 strains. Vertical line at a log-odds of 4 indicates a strict threshold for predicting a gene as defensive. (**B**) Left: number of protein clusters belonging to one of four categories of predicted defense genes. Right: examples of the types of predicted defense genes. Shaded areas represent regions of homology between genes, with darker regions representing stronger sequence similarity. REase, restriction endonuclease; MTase, methyltransferase. (**C**) Line plot showing the cumulative number of uncharacterized, predicted defense proteins below the indicated coverage cutoffs. (**D**) Frequency of domain families found in uncharacterized, predicted defense clusters. RHH, ribbon-helix-helix; HTH, helix-turn-helix. (**E**) Histogram of the number of genomes each protein cluster is encoded by. Protein clusters encoded by five or fewer genomes are considered part of the accessory pangenome and clusters encoded by all genomes are considered part of the core pangenome. (**F**) Frequency of genes residing in defense islands (±10 genes of a known defense gene), prophages, plasmids, or some combination thereof for all, known defense, or uncharacterized, predicted defense genes. Solid circles indicate the type of element genes reside in. Error bars represent the 95% confidence interval after resampling the 69 genomes 200 times.

Next, we used DefensePredictor to identify defense proteins encoded in a set of 69 diverse *E. coli* strains, primarily from the ECOR (*E. coli* reference) collection (22), which captures much of the pangenome diversity of this species (table S5). Using a stringent log-odds cutoff of 4, the model predicted that 1,991 out of ~321,000 genes from these strains encode defense proteins (Fig. 2A). Clustering at 80% reciprocal coverage and 30% identity revealed 624 protein clusters that were predicted as defensive. In contrast, DefenseFinder identified only 152 protein clusters that were part of a complete system across all strains.

Of the 512 protein clusters identified by DefensePredictor but not DefenseFinder, 219 were homologous to a known defense protein, but in a new context, meaning they were not adjacent to genes typically found in the same operon (Fig. 2B). By running three iterations of the sensitive alignment tool HHblits (23), we saw that an additional 103 protein clusters were homologous to known defense proteins with greater than 40% coverage. Using ESMFold to predict protein structures (13) and Foldseek to perform a structural search (24), we found that an additional 22 protein clusters had structural homology to a known defense protein.

Of the remaining 168 protein clusters, 65 aligned to a known defense protein with less than 40% coverage (Fig. 2C), and 103 had no detectable homology with a known defense protein. We will refer to these proteins with less than 40% coverage (including no coverage) as ‘uncharacterized’ systems as the lack of, or limited homology, makes inference of their defense functions difficult or uncertain (table S5). We allow for some homology in this category because defense proteins can have regions of homology but distinct mechanisms of defense. For example, Cas9 (25) and KpnI (26) share an HNH nuclease domain, but they recognize their phage targets via distinct mechanisms. Similarly, the DRT (defense-associated reverse transcriptase) (27, 28) and retron systems (29) each feature a reverse transcriptase but provide defense in substantially different ways.

We cataloged the domains of the protein clusters comprising the uncharacterized systems using HHblits to query the protein family (Pfam) database. Of the 168 protein clusters, 83 (49%) contained a domain previously implicated in defense (table S5). These protein clusters were not categorized as homologs of known defense proteins because they had less than 40% coverage with a known defense protein. The three most common domain families were PD-(D/E)XK nucleases, P-loop NTPases, and a ribbon-helix-helix (RHH) DNA binding domain, found in 9%, 5%, and 4%, respectively, of protein clusters within the uncharacterized set (Fig. 2D). Notably, 51% of the predicted defense protein clusters had no detectable domains in common with known defense proteins.

Next, we investigated the genomic distribution and genomic context of the uncharacterized defense proteins. We found that 89% of these proteins had homologs encoded by five or fewer genomes in our *E. coli* strain collection, compared with 70% of known defense proteins, and 52% of all proteins (Fig. 2E). The enrichment of these uncharacterized defense genes in the accessory genome suggests that they are often mobile and horizontally transferred, like known defense genes (30, 31).

To examine the frequency with which the uncharacterized, predicted defense genes reside in mobile genetic elements, we first predicted prophages and plasmids in our *E. coli* collection using geNomad (32). Considering all proteins individually (*i.e*., no longer grouping by protein clusters), we found that 17% of the uncharacterized defense genes reside in plasmids, compared with 26% of known defense genes and 8% of all genes (Fig. 2F). Similarly, we found that 21% of uncharacterized defense genes reside in predicted prophages, compared with 15% of known defense genes and 6% of all genes. Thus, the uncharacterized defense genes identified reside in mobile elements well above background levels, similar to known defense genes. Finally, we found that 14% of uncharacterized defense genes reside in defense islands (defined as within 10 genes of a known defense gene) compared with 17% of known defense genes and 2% of all genes. Thus, predicted, but as-yet uncharacterized defense genes are enriched in defense islands, similar to known defense genes. Overall, the patterns of genomic distribution and context for our predicted defense genes resemble that of known defense genes.

## Predicted defense systems provide protection against phage

To assess whether the predicted defense genes not identified by DefenseFinder confer anti-phage defense, we selected 94 putative transcriptional units (TUs) for experimental validation, which included 13, 8, and 73 systems from the remote homolog, structural homolog, and uncharacterized categories in Fig. 2B (table S6). We randomly selected 38 of these TUs for validation, whereas the other 56 were selected based on properties of interest, such as protein domains not previously validated in defense, novel domain combinations, or particularly high probabilities of defense. We defined TUs by taking groups of consecutive genes, transcribed in the same direction, and each separated by 30 base pairs (bps) or less. TU boundaries were then adjusted based on manual inspection. For the 31 TUs we selected with multiple genes, 18 (58%) had all genes predicted as defensive and 13 (42%) had at least one gene that was not predicted defensive.

**Fig. 3.**
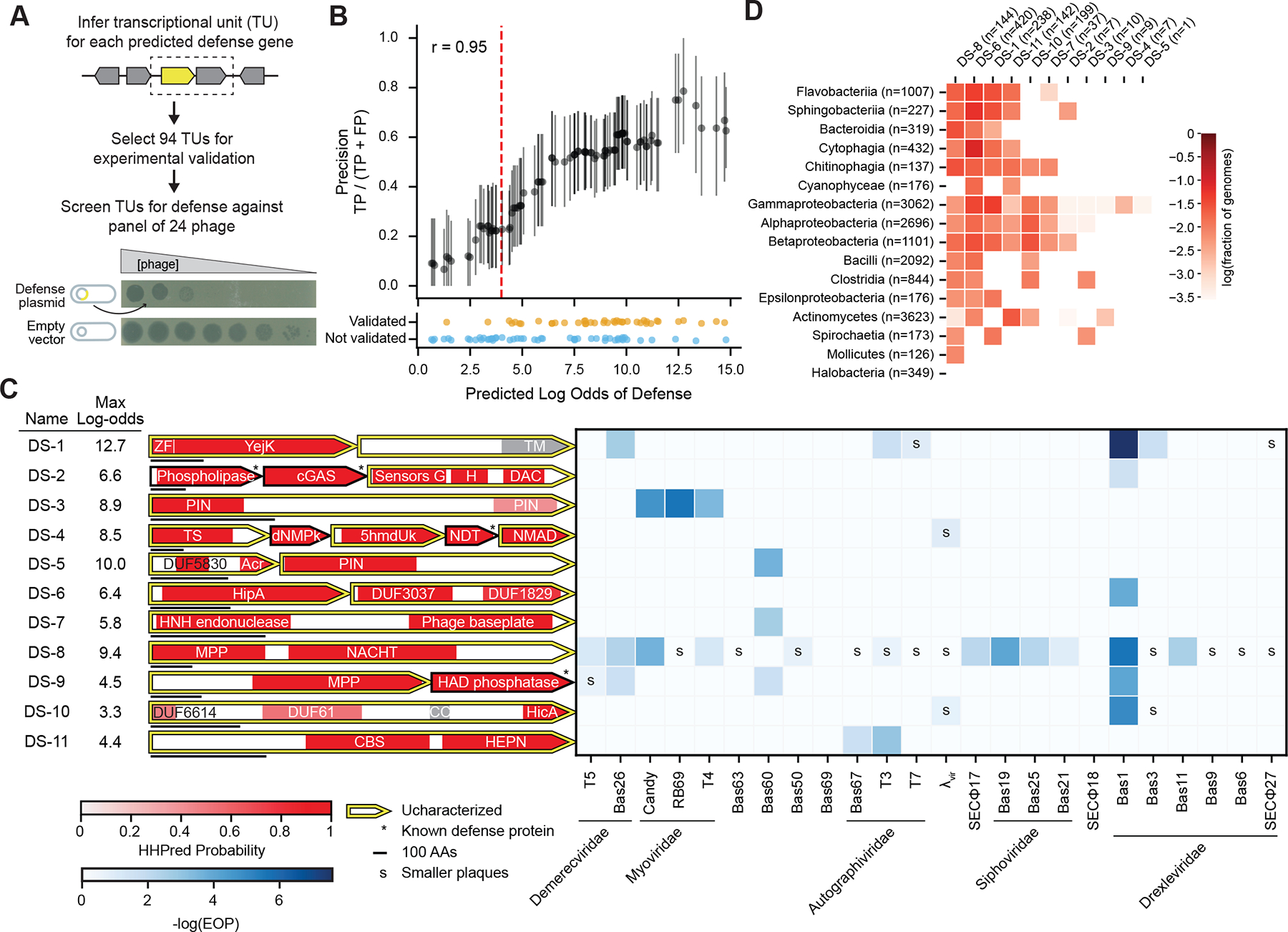
Predicted defense systems validate experimentally at a high rate. (**A**) Pipeline for validating predicted defense systems. The yellow gene represents an uncharacterized, predicted defense gene. The boxed genes represent a putative transcriptional unit. An example plaquing assay is shown, with phage plaques forming on a lawn of *E. coli*, at decreasing concentrations of phage. (**B**) Bottom: each point represents one predicted TU. TUs are plotted by their log-odds of defense, and colored by whether they provided protection. Top: validation rate at increasing log-odds thresholds, estimated by taking all systems ±2 log-odds of each point. Error bars represent 95% confidence intervals after resampling TUs 1,000 times. Pearson correlation between the log-odds and estimated validation rate is indicated. Red dashed line represents a log-odds cutoff of 4. TP, true positive; FP, false positive. (**C**) Left: domain annotations for validated defense systems containing domains with a defined function that had not previously been validated in defense. Horizontal bar underneath each system represents 100 amino acids. Genes with an asterisk represent homologs of known defense genes. Uncharacterized defense proteins are outlined in yellow. The name and maximum log-odds of each system is indicated. Right: defense heatmap with EOP differences indicated by blue squares and plaque size differences indicated by the letter “s”. Phages are ordered by taxonomy. ZF, zinc finger; DAC, di-adenylate cyclase; TS, thymidylate synthase; dNMPk, dNMP kinase; 5hmdUk, 5-hmdU DNA kinase; NMAD, nucleotide-modification associated domain; MPP, metallophosphatase; TM, transmembrane; CC, coiled-coil; AA, amino acid. (**D**) Taxonomic distribution of systems with novel domains. The frequency of each system having homologs across the 16 most abundant prokaryotic classes is indicated by red squares.

To test for anti-phage defense, we placed each TU with its predicted native promoter region on a low-copy number plasmid in *E. coli* MG1655 and challenged these strains with a panel of 24 diverse *E. coli* phages (Fig. 3; fig. S2). In total, 42 (45% of 94) of the cloned TUs produced smaller plaque sizes or reduced the efficiency of plating (EOP) at least ten-fold relative to an empty vector control strain, including 32, 3, and 7 TUs from the uncharacterized, structural homolog, and remote homolog categories, respectively (Fig. 3B; table S7). We refer to these validated TUs as DefensePredictor discovered systems (DSs), with genes in multi-gene TUs denoted by an alphabetical suffix, e.g., *DS-8A* is the first gene of DS-8.

To estimate the experimental validation rate at different predicted thresholds of defense, we first defined the log-odds of each TU as the maximum log-odds of its constituent genes. We then calculated the validation rate for groups of TUs that were within four log-odds of each other. We observed a strong correlation between the validation rates and predicted log-odds of defense *(r* = 0.95; Fig. 3B), suggesting that DefensePredictor can effectively discriminate between defensive and non-defensive TUs.

To begin elucidating the function of the 42 validated TUs, we further annotated their protein domains (see Methods; table S8). This analysis identified five Pfam domains of unknown function (DUF) that were newly implicated in defense: DUF3037, DUF1829, DUF5830, DUF6988, and DUF6650. We also found 10 domains with a defined function that had not previously been validated in defense, including a YejK nucleoid-associated domain, a di-adenylate cyclase, a PIN nuclease, a 5-hydroxymethyluracil kinase, a thymidylate synthase, a HipA-family kinase, a phage baseplate protein, a metallophosphatase, a HicA mRNA interferase, and a CBS adenosyl-binding/dimerization domain (Fig. 3C). Note that a PIN domain and a HipA kinase were recently validated elsewhere (10, 33–35), and the HicA domain was predicted as defensive in a bioinformatic study (36), but none of these systems were included in the training set, making them novel from the model’s standpoint. We searched for homologs of the 11 systems that contain these newly validated defense domains in the ~17,000 representative genomes and found that 9 had homologs in at least two bacterial classes (Fig. 3D), indicating that these systems, which likely function via novel mechanisms, are broadly distributed in bacteria.

**Fig. 4.**
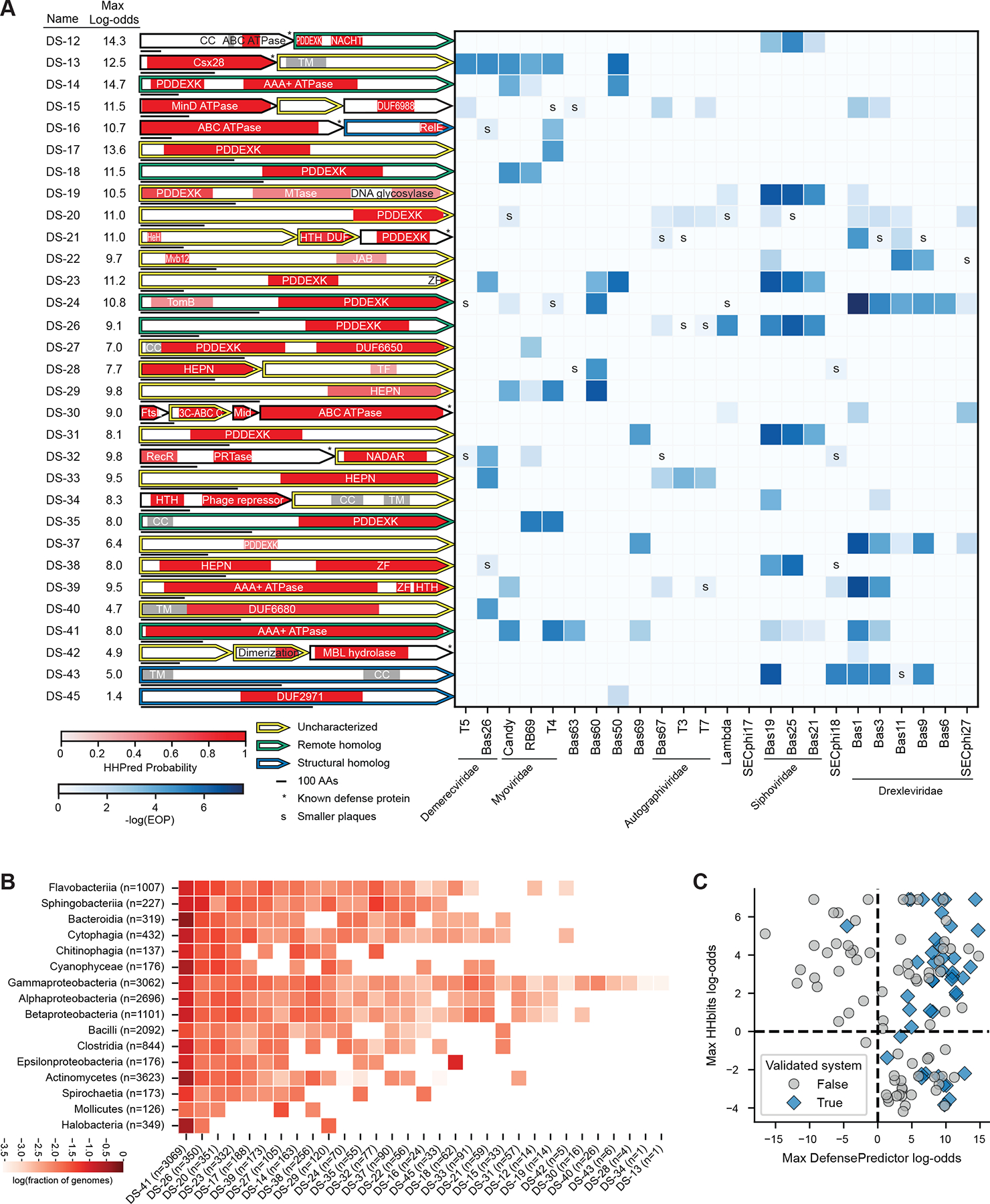
Validated systems contain diverse domains in diverse contexts. (**A**) Defense heatmap (as in Fig. 3C) for validated systems with known defense domains or domains without a known function. Proteins are outlined using the same color scheme as Fig. 2C, where only the proteins that are most distant from known defense proteins are colored. MTase, methyltransferase; HTH, helix-turn-helix; HeH, helix-extension-helix; TPR, tetratricopeptide repeat; TF, transcription factor; PRTase, phosphoribosyltransferase. (**B**) Taxonomic distribution of systems from panel (**A**), presented as in Fig. 3D. (**C**) Scatterplot showing the maximum log-odds of homology to known defense proteins using HHblits versus maximum log-odds of defense using DefensePredictor for all cloned TUs. The shape and color of each TU indicates whether it provided anti-phage defense. Dashed lines are drawn at a log-odds of zero for each axis.

For the 31 additional defense systems that we validated, 13 have PD-(D/E)XK nuclease domains, 7 have P-loop NTPase domains (e.g. AAA+, NACHT, ABC, etc.), and 4 have HEPN nuclease domains (Fig. 4A), though each was embedded within a protein or operon that distinguished it from known defense systems that also harbor these domains. Of these 31 systems, 24 had homologs outside the proteobacteria, with three systems (DS-41, DS-26, and DS-29) even present in the archaeal Halobacteria (Fig. 4B). Thus, many of these systems have homologs that likely provide protection in a broad range of species.

## DefensePredictor is more sensitive and precise than remote homology detection

Although systems with domains not previously validated in defense represent the most obvious source of novelty, systems with known defense domains have the potential to function via novel mechanisms. For example, the predicted structures of the 14 PD-(D/E)XK nuclease proteins we identified align well with the catalytic region of the restriction enzyme EcoRI (37) (figs. S3A–B), but there is extensive structural diversity, and often entirely separate domains, outside this catalytic core (figs. S3C, S4). Similar observations were made with other classes of proteins, including those harboring HEPN domains and NADAR domains (figs. S5A–E, S6A–I).

Because some of our validated systems had distant homology to known defense systems, we wondered whether DefensePredictor was simply detecting remote homology. However, not all homologs of known defense systems were predicted to be defensive, suggesting that the model can somehow discriminate between defensive and non-defensive proteins that share homology with known defense proteins. To test this idea further, we randomly selected 24 TUs that have remote homology to known defense proteins based on three iterations of HHblits, but have a predicted log-odds of defense less than zero (table S6). These TUs included six PD-(D/E)XK nucleases, the most prevalent domain in our validated hits. When we challenged these 24 TUs with our panel of phages, only one provided anti-phage defense (fig. S7A).

Considering all 118 TUs that we cloned and tested (the 94 selected in Fig. 3 based on high log-odds scores and the 24 selected based on homology but low log-odds scores), relying exclusively on HHblits would have correctly identified 32 validated systems at a log-odds cutoff of 0, achieving a precision of 40% and recall of 74% (Fig. 4C). DefensePredictor achieved a higher precision of 45% and recall of 98% at a log-odds cutoff of 0 (p = 0.13, one-sided permutation test for precision and p < 0.01, one-sided McNemar test for recall). Considering a more continuous range of cutoffs, DefensePredictor consistently had higher precision than HHblits across recall cutoffs (Fig. S7B), achieving an AP of 0.62, compared with an AP of 0.42 for HHblits. Collectively, our results demonstrate that DefensePredictor outperforms simple homology detection and can distinguish between defensive and non-defensive members of a given protein family.

## Newly validated defense domains are essential for protection

**Fig. 5.**
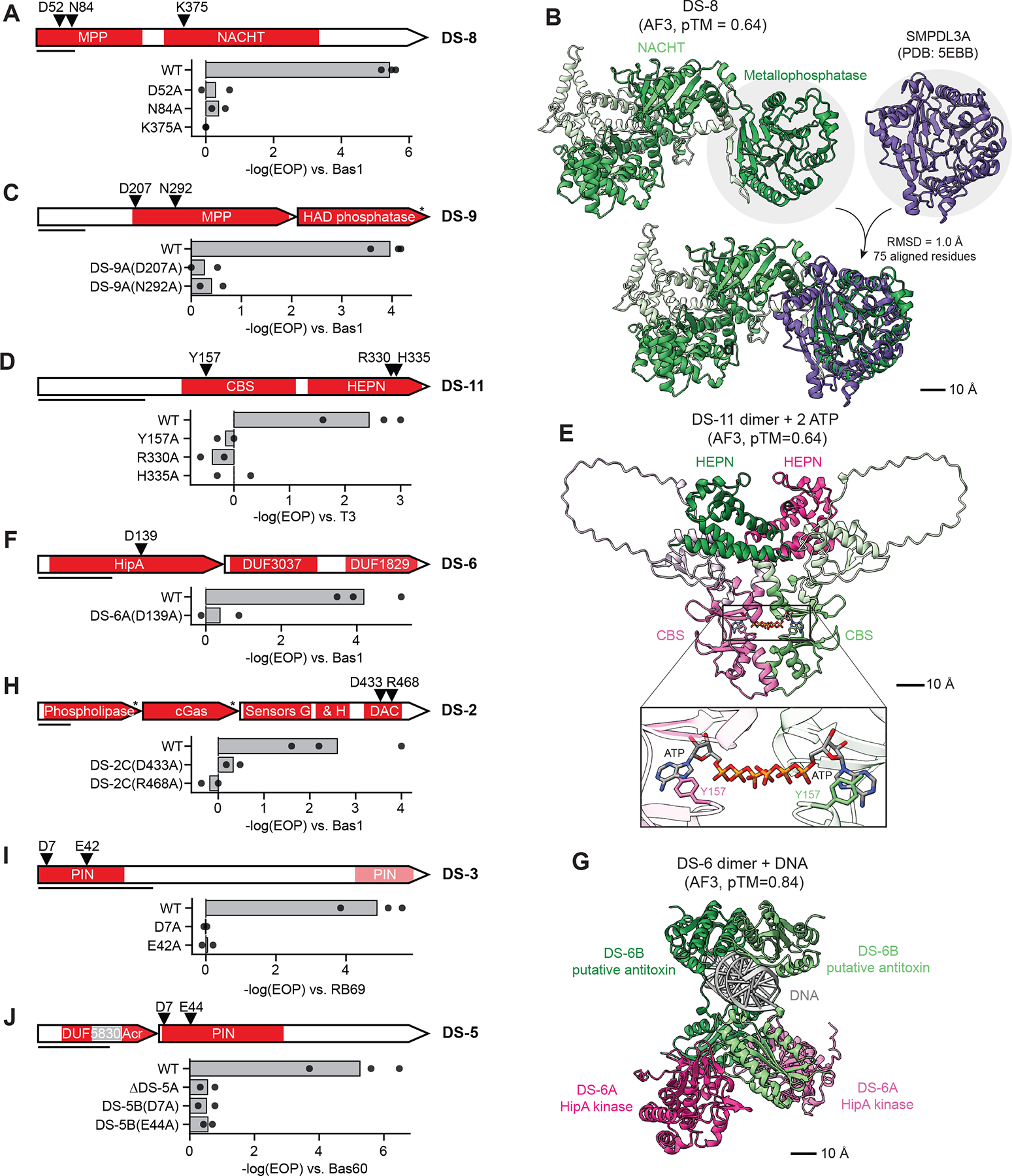
Newly validated defense domains are essential for protection. (**A, C, D, F, H-J**) For the systems DS-8 (**A**), DS-9 (**B**), DS-11 (**D**), DS-6 (**F**), DS-2 (**H**), DS-3 (**I**), DS-5 (**J**), the domain architecture is shown at the top along with the location of catalytic mutants tested. Bar graphs show −log(EOP) versus the phage indicated for the wild-type system and variants harboring the mutations indicated. Bars represent average values and points represent individual replicates. (**B**) Left: Predicted structure of DS-8 with domains highlighted in dark green. Right: solved structure of SMPDL3A (PDB: 5EBB). Bottom: Alignment between DS-8 and SMPDL3A. Scale bar is shown. (**E**) Top: predicted structure of a DS-11 dimer with two ATP molecules. One copy of DS-11 is shown in green and a second copy in pink. Protein domains are shown as dark regions. Bottom: predicted interaction between DS-11 protomers and ATP. The Y157 residues are shown for each protomer. Scale bar is shown. (**G**) Predicted structure of two copies of DS-6A (pink), two copies of DS-6B (green), and DNA. Scale bar is shown.

To gain insight into the functions of new systems identified by DefensePredictor, we further investigated several that contain domains not previously validated in defense. First, we investigated DS-8, a single protein system containing a metallophosphatase domain (Fig. 5A) which is homologous to the human protein SMPDL3A, a phosphodiesterase that cleaves cGAMP to modulate cGAS-STING immunity signaling (38). The AlphaFold3-predicted structure of DS-8 (figs. S8A–B; table S9) closely aligned with the solved structure of SMPDL3A (39) (Fig. 5B). DS-8 also contains a NACHT domain (a type of P-loop NTPase), a domain found in other defense systems (6, 40, 41) where they typically facilitate oligomerization and recognition of phage proteins, leading to activation of an N-terminal effector. Mutating either of two predicted catalytic residues in the metallophosphatase domain or a residue likely critical to NACHT ATPase activity completely ablated defense by DS-8, indicating that both domains and metallophosphatase activity are essential for protection (Fig. 5A; table S7). The identity of the cyclic nucleotide degraded by DS-8 remains to be identified, but this system highlights a role for metallophosphatases in anti-phage defense and, given the homology to SMPDL3A, reveals a connection between prokaryotic and human immunity.

The system DS-9 has two genes, the first harboring a metallophosphatase domain homologous to that of DS-8, and the second a predicted haloacid dehalogenase-like (HAD) phosphatase (Fig. 5C). HAD phosphatases are an enormous, but poorly understood family of enzymes (42). This domain has been identified in other defense systems (9, 43), but its function in defense remains unknown. When we mutated the predicted catalytic residues in the metallophosphatase domain of DS-9A, we saw a loss of defense, suggesting it is essential for protection (Fig. 5C). As with DS-8, the target of the metallophosphatase remains unknown, as does that of the HAD phosphatase, but these two systems demonstrate that novel enzymes and novel signaling molecules likely remain to be discovered in antiphage defense.

Next, we examined DS-11, a one-protein system containing CBS and HEPN domains (Fig. 5D). CBS domains facilitate dimerization of some enzymes by binding adenine derivatives including AMP, ATP, and Ap4A (44, 45). There are no characterized defense systems with CBS domains, though these domains are prevalent in bacteria, archaea, and eukaryotes. HEPN domains are typically dimeric ribonucleases (46), including in known defense systems such as Cas13 (8, 47). When we predicted the structure of two copies of DS-11 with two ATP molecules, AlphaFold3 predicted a reasonably strong interaction between protomers (ipTM (interface predicted Template Modeling score) 0.59) and strong interactions between each protomer and ATP (ipTM 0.88) (Fig. 5E; figs. S8C–D). The predicted structure revealed a likely interaction between Y157 of the CBS domain of DS-11 and the adenine base of ATP; when we substituted Y157 with alanine, DS-11 no longer defended against phage (Fig. 5D). Defense was also ablated when we mutated catalytic residues in the HEPN domain. Together, these data suggest that the CBS domain of DS-11 regulates HEPN nuclease activity by binding an adenine derivative that remains to be discovered.

The two protein system DS-6 features DS-6A, a predicted serine/threonine kinase related to the toxin HipA (48), and a second protein, DS-6B, with DUF3037 and DUF1829 (Fig. 5F) with no similarity to HipA’s cognate antitoxin HipB. In *E. coli* MG1655, HipA phosphorylates glutamyl-tRNA synthase to block translation (49) unless neutralized by HipB, which also binds and represses the *hipAB* promoter (50). We independently discovered DS-6 (renamed HipAD) recently (35), but it was not included in our model’s training set so it still highlights the ability of DefensePredictor to find uncharacterized systems. To determine whether DS-6B interacts with DS-6A and with DNA akin to HipB, we used AlphaFold3 to fold two copies of DS-6A with two copies of DS-6B, mimicking the stoichiometry of HipAB, and DNA (figs. S8E–F). AlphaFold3 predicted strong interactions between DS-6A and DS-6B (ipTM 0.76–0.79), and slightly weaker interactions between DS-6B and DNA (ipTM 0.67) (Fig. 5G). When we mutated a key predicted catalytic residue in DS-6A, we observed a loss of defense, suggesting that kinase activity is essential for protection. Thus, we hypothesize that DS-6 functions as a toxin-antitoxin (TA) system akin to HipAB, with the kinase activity of DS-6A providing protection against phage, possibly via an abortive infection mechanism similar to other TA-based defense systems (51, 52).

The system DS-2 has three genes: a phospholipase, a cyclic GMP-AMP (cGAMP) synthase (cGAS), and a diadenylate cyclase (DAC) (Fig. 5H). The phospholipase and cGAS together constitute an apparent CBASS system (53) in which the cGAS presumably senses phage, triggering cGAMP synthesis, which activates the phospholipase to degrade the cell membrane and abort infection. An association between CBASS and DACs was recently noted bioinformatically (54), but DACs have not been validated in phage defense. Using webFlaGs (55), we found that 15 of 30 homologs of the DAC identified here were co-located with a CBASS (fig. S9), confirming their association and suggesting they function together. To determine whether the DAC is essential for DS-2-based defense, we mutated two of its predicted catalytic residues. In each case, defense was eliminated. Thus, DS-2 may represent a novel type of CBASS system that employs two different cyclase genes.

DS-3 is a one-protein system with a PIN ribonuclease domain split between its N and C-terminus (Fig. 5I, figs. S10A–C). PIN domains are best characterized in VapC, the toxic component of VapBC TA systems, where they function to cleave tRNAs (56). When we mutated catalytic residues in DS-3, the system no longer defended against phage, suggesting this domain is essential for protection. There is no cognate antitoxin for DS-3, suggesting that it may be auto-inhibited until phage infection occurs.

Finally, DS-5 is a two protein system that also features a PIN ribonuclease, though not closely related to that of DS-3, along with a second protein containing a DUF5830 and a domain homologous to the anti-CRISPR protein AcrIF4 (57). Mutating the catalytic residues in the PIN domain eliminated defense. Deleting the AcrIF4 domain-containing gene also ablated defense by DS-5 but did not lead to unrestrained toxicity of the ribonuclease indicating that DS-5 is likely not a TA system and that both components are essential to defense (Fig. 5J). Altogether, the systems probed here illustrate how DefensePredictor can identify novel anti-phage defense systems whose further study will likely reveal new mechanisms of immunity.

## Thousands of predicted defense genes remain to be validated in *E. coli* and beyond

**Fig. 6.**
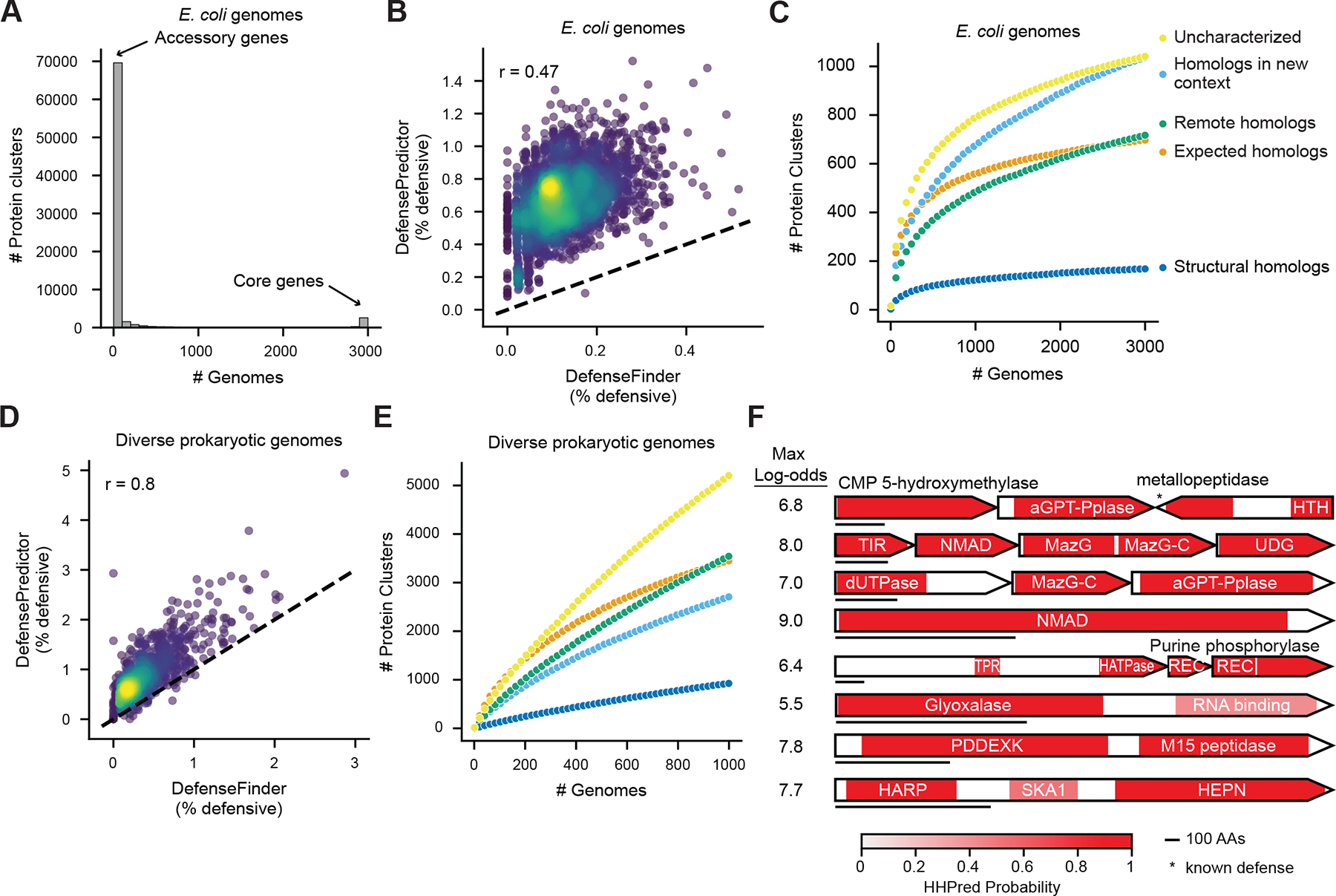
Thousands of predicted defense proteins remain to be validated. (**A**) Histogram of the number of genomes each protein cluster is encoded by in a set of 3,000 *E. coli* strains. (**B**) Scatterplot showing the percent of genes per genome dedicated to defense as defined by DefenseFinder or DefensePredictor for 3,000 *E. coli* strains. The dashed line represents the function *y* = *x*. Pearson correlation is indicated. (**C**) Average number of unique protein clusters for different categories of predicted defense proteins (see Fig. 2B) when considering between 1 and 3,000 *E. coli* genomes in steps of 50. Averages were calculated after resampling genomes 10 times at each step. (**D**) Same as (**B**) for 1,000 diverse prokaryotic strains. (**E**) Same as (**C**) for 1,000 diverse prokaryotic strains. Colors are the same as (**C**). (**F**) Domain annotations for predicted defense systems containing domains that have not been validated as defensive. Horizontal bar underneath each predicted system represents 100 amino acids. Genes with an asterisk represent homologs of known defense genes. The maximum log-odds of each predicted system is indicated. aGPT-Pplase, alpha-putrescinyl/glutamylthymidine pyrophosphorylase; HTH, helix-turn-helix; NMAD, nucleotide modification associated domain; UDG, uracil DNA glycosylase; TPR, tetratricopeptide repeat; HATPase, histidine kinase-like ATPase; REC, receiver domain; SKA1, spindle and kinetochore associated protein.

We sought to assess the landscape of defense systems in the broad *E. coli* pangenome, beyond the initial set of 69 strains examined. We therefore applied DefensePredictor to a large, diverse set of 3,000 *E. coli* and *Shigella* genomes. We clustered the proteins encoded by these genomes at 30% sequence identity and 80% reciprocal coverage, and saw that most genes reside in the accessory pangenome, with 78% present in 10 strains or fewer (Fig. 6A). We ran DefensePredictor on all 3,000 strains and found an average of 32 defense genes per genome, compared with an average of 6 for DefenseFinder (Fig. 6B). We found that DefensePredictor made similar predictions for homologs, where 85% of clusters with at least one predicted defense protein had a majority of proteins in the cluster predicted as defensive (fig. S11B). When we plotted the number of unique protein clusters containing at least one predicted defense protein against the number of genomes included in our analysis (Fig. 6C), for each category of predicted defense (Fig. 2B), there was a non-saturating increase, with a total of 1,041 protein clusters harboring a predicted, uncharacterized defense protein (table S10). Updated versions of DefenseFinder and PADLOC (58), another homology-based identification tool, identified 200 (19%, including 123 proteins that aligned to defense proteins from this work) and 28 (3%), respectively, of these unique proteins. Together, our results indicate that the *E. coli* pangenome is vast and still incompletely defined.

To explore the defense landscape outside of *E. coli*, we analyzed the 1,000 randomly selected RefSeq genomes that we ran DefensePredictor on above. The ~741,000 unique protein clusters encoded by these 1,000 genomes (figs. S11B–C) far exceeded the ~77,000 unique protein clusters encoded by the 3,000 *E. coli* strains. DefensePredictor identified an average of 33 defense genes per genome, compared with an average of 14 for DefenseFinder (Fig. 6D). When we plotted the number of unique predicted defense proteins against the number of genomes included in our analysis, we again saw a non-saturating increase (Fig. 6E), with a total of 5,204 protein clusters harboring a predicted uncharacterized defense protein, including 3,140 with no detectable homology to a known defense protein using three iterations of HHblits (table S4). We identified 568 domains across these clusters that have not previously been validated as defensive, including 244 DUFs. While some of these domains may represent false positives, the majority represent promising candidates for future study.

To illustrate some interesting predicted defense domains, we selected eight predicted systems to highlight in Fig. 6F. These systems are sometimes found in defense islands (figs. S12–13). The uncharacterized defense domains in these systems include an alpha-putrescinyl/glutamylthymidine pyrophosphorylase (aGPT-Pplase), a NTP pyrophosphohydrolase (MazG), a predicted nucleotide kinase (MazG-C), a dUTPase, a nucleotide modification associated domain of unknown function, and a purine phosphorylase domain, which are all implicated in modifying nucleotides. Several of these domains were also predicted to function in defense in a bioinformatic study (59), but none have been experimentally validated. We also identified several domains unrelated to nucleotide metabolism: a glyoxalase, an M15 family peptidase, and a DNA-dependent ATPase (HARP) domain. These results suggest that DefensePredictor will enable the discovery of scores of additional systems in diverse prokaryotes.

## Discussion

DefensePredictor takes as input the sequence of a gene and its genomic neighbors, and predicts whether this gene functions in anti-phage defense. When applied to a set of 69 diverse *E. coli* strains, we identified >600 protein clusters that were predicted to be defensive, including 154 with no detectable homology to or less than 40% coverage with any known defense protein. We cloned 94 TUs that were predicted to be defensive and that were not identified by DefenseFinder, achieving an experimental validation rate of 45% with a strong correlation between the predicted log-odds of defense and the experimental validation rate. The new systems we identified indicate that *E. coli* harbors a much broader landscape of anti-phage defense than previously appreciated, expanding the likely number of systems by multiple orders of magnitude. The new systems feature domains not previously validated in defense, including homologs of eukaryotic innate immune proteins.

Although some predicted defense systems did not validate and could represent false positives, we anticipate that many will validate if challenged with a larger, more diverse panel of phages. Further, some systems may not be adequately expressed in *E. coli* K-12 strains and may function in a different host strain. Finally, detecting the defense function of some systems may require redefining the boundaries of the system to capture additional genes or regulatory elements.

By analyzing DefensePredictor’s outputs for 100 systems deposited in DefenseFinder after we built the dataset for DefensePredictor, we estimate that the model has a false negative rate between 18% and 32%, depending on the cutoff used. The model’s false negative rate can likely be improved in future models by including in a new training set the dozens of systems discovered here and elsewhere since the start of this work. New language models (e.g. see refs (60–62)) may also improve model performance. Notably, DefensePredictor cannot directly identify defensive non-coding RNAs (e.g. CRISPR arrays or retron non-coding RNAs) since its primary input is protein sequences, so new genomic language models may enable the discovery of novel defensive of non-coding RNAs. DefensePredictor did not predict all of the genes identified strictly through homology searches, *e.g*. via DefenseFinder, suggesting that both approaches, and experimental screening, will be needed to comprehensively catalogue the repertoire of defense systems in a strain of interest. Finally, we note that the training set used for DefensePredictor features a predominance of systems identified or validated in *E. coli*, so whether the model performs as well on other species, particularly those distantly related to *E. coli*, remains to be tested.

Application of DefensePredictor to a set of 3,000 *E. coli* and 1,000 diverse prokaryotic genomes indicated that the number of predicted systems does not saturate. This finding underscores both the enormous genetic diversity of even a single species of bacteria like *E. coli* and the vast number of anti-phage defense systems that remain to be found. As an open-source software tool that can be run on any prokaryotic genome of interest, we anticipate that DefensePredictor will help catalyze the mapping of such defense systems and the discovery of novel molecular functions, while also helping to further elucidate connections between prokaryotic and eukaryotic immunity.

## Supplementary Material

Supplementary Materials

Table S6

Table S10

Table S5

Table S7

Table S11

Table S8

Table S2

Table S4

Table S9

Table S3

Table S1

Materials and Methods

Figs. S1 to S13

Tables S1 to S11

References (65–91)

## Data Availability

All data are available in the main text or the supplementary materials. All custom scripts are available on Zenodo (63). DefensePredictor is available at https://github.com/PeterDeWeirdt/defense_predictor and archived on Zenodo (64). All materials are available upon reasonable request to the corresponding author.
