# Supplementary material for: DefensePredictor: A Machine Learning Model to Discover Prokaryotic Immune Systems
Source: Science. Author manuscript; Available in PMC 2026 Apr 20. (PMC13092281; doi:10.1126/science.adv7924)
Supplement: Supplementary Materials [file NIHMS2163519-supplement-Supplementary_Materials.pdf]

Science

AAAS

Supplementary Materials for

DefensePredictor: A Machine Learning Model to Discover Prokaryotic Immune Systems

Peter C. DeWeirdt, Emily M. Mahoney, Michael T. Laub

Corresponding author: laub@mit.edu

The PDF file includes:

Materials and Methods
Figs. S1 to S13
Titles of tables S1 to S11

Other Supplementary Materials for this manuscript include the following:

Tables S1 to S11

Materials and Methods

Compute resources

Intel Xeon Platinum 8260 or Xeon Gold 6248 computers with Nvidia Volta V100 GPUs from MIT's Supercloud (*65*) were used for computational analyses, unless otherwise noted.

Dataset assembly

17,454 assembled genomes were downloaded from RefSeq (*66*) on May 2, 2023, comprising all bacterial, archaeal, and viral representative and reference genomes. Genomes were searched for defense systems using DefenseFinder version 1.2.2, and all genes that were identified as part of a full system were labeled as our positive gene set.

To construct the negative gene set, GO Processes were extracted from the General Feature Format (GFF) table for each genome. GO processes were deemed non-defensive if more than 90% of gene members were not identified by DefenseFinder. Only seven GO processes did not meet this threshold: 'maintenance of CRISPR repeat elements', 'DNA modification', 'DNA methylation', 'DNA restriction-modification system', 'defense response to virus', 'nucleic acid phosphodiester bond hydrolysis', and 'DNA methylation on adenine'. Genes belonging to COGs of mobile elements as defined by Marakova et al. (*4*) were added to the negative set as well. Finally, homologs of secretion system effectors from BastionHub (*67*) were identified using MMseqs2 with coverage and identity cutoffs of 90% and added to the negative gene set. Genes were removed from the negative set if they were identified by DefenseFinder or if they were part of a TA system as indicated by their name containing one of the phrases 'toxin-antitoxin', 'addiction module', or 'abortive infection.'

To reduce redundancy, all proteins were clustered at 30% sequence identity and 80% reciprocal coverage, using the MMseqs2 cluster algorithm with the parameters `–c 0.8 –cov-mode 0 –min-seq-id 0.3 –cluster-mode 0 –s 6`. One protein was selected per cluster.

To assess the model's ability to predict defense proteins that are not homologs of known ones, we divided our dataset into five folds, ensuring that groups of homologs remained together in each fold. To identify homologs more sensitively than the MMSeqs2 clustering above, we performed an all-by-all MMseqs2 profile search. To build profiles for the proteins (both control and defense proteins) in our dataset, we queried them against a non-redundant set of proteins from our Refseq reference genomes using the parameters `–s 6 –num-iterations 3`. We then searched these profiles against all proteins in our dataset using the parameter `–s 7.5`. We connected two proteins if at least one had greater than 30% identity and 80% coverage (not necessarily reciprocal as above). We then clustered this homology network using the Louvain algorithm in networkx (*68*). For defense proteins, to ensure even distant homologs were grouped together, we further clustered these proteins based on the HMM they aligned to. We clustered defense proteins matching homologous HMM profiles, defined as profiles that aligned to at least one of the same proteins in the set of reference genomes. Furthermore, to prevent our model from memorizing the exact context of a defense protein, we clustered defense proteins that aligned to HMM profiles that were considered exchangeable by DefenseFinder (e.g. non-homologous CBASS effectors are exchangeable). We then split clusters (both control and defense) into five folds using the `GroupKFold` splitter from scikit-learn (*69*), attempting to balance the number of functional groups (GO processes, COGs, and defense HMMs) represented in each fold while keeping clusters together.

Model training

To generate ESM2 embeddings for the feature matrix the 150 million parameter ESM2 model (*70*), 'esm2_t30_150M_UR50D', was used. The feature matrix was then assembled as described in the Results. The LightGBM (*16*) framework was used to train a gradient boosting classifier for each split. Hyperparameters for each classifier were fit with optuna (*17*), splitting data from the non-test folds into training and validation sets using the same splitting procedure as above. The number of leaves for the model was fit between 4 and 256, and the minimum number of child samples was fit between 32 and 1,024 for 15 trials. We set the learning rate to 0.01 and number of estimators to 30,000 for training. Training was stopped after the average precision on the validation data did not increase for 200 iterations.

Calculation of precision and recall

To calculate the precision and recall of each modeling approach, test genes were ranked by probability, p-value, or cosine similarity for DefensePredictor, guilt-by-association, and ESM2, respectively. Genes were ranked by bit-score for Foldseek/ProstT5, BLAST, and HMM search. For nearest-neighbor approaches, we ranked genes by their negative score if their nearest neighbor was non-defensive. To prevent these metrics from being dominated by the largest clusters of defense genes or the largest GO process or COGs, we selected the top prediction per functional group for each approach. Then, for all modeling approaches at descending predicted thresholds of defense, we calculated $\text{Precision} = \frac{\text{\# True Positives}}{\text{\# True Positives} + \text{\# False Positives}}$, and $\text{Recall} = \frac{\text{\# True Positives}}{\text{\# True Positives} + \text{\# False Negatives}}$. Average precision was calculated as $\text{AP} = \sum_n (\text{Recall}_n - \text{Recall}_{n-1}) \times \text{Precision}_n$, across all thresholds n. Precision-recall curves were averaged across all five folds by estimating each model's precision at shared recall cutoffs. Precision was estimated by taking the precision at the first observed recall cutoff greater than or equal to the shared recall cutoff.

Guilt-by-association implementation

To implement a guilt-by-association approach, all proteins in the set of representative genomes that belong to an MMseqs2 cluster containing at least one protein in the dataset (positively or negatively labeled) were extracted. For each gene that codes for one of these proteins, whether it resides within 10 genes of a DefenseFinder-identified defense gene was assessed. In making this assessment, neighboring defense genes belonging to the same fold as the query gene or that were part of a known system with the query gene were discarded. Then for each MMseqs2 cluster, the significance of its association with known defense genes was calculated using a one-sided Fisher's exact test, where the frequency that each MMseqs2 cluster resided within 10 genes of a defense gene was compared with random expectation. All test genes were ranked based on the significance of their cluster's association.

SHAP value calculation

The contribution of each feature to the final output prediction of genes in held-out folds was calculated using the shap package (*71*). To identify the most important individual features, SHAP values were aggregated for each feature across all true positive genes in held-out folds

using a weighted average, such that HMM clusters contributed equally to each feature's average. The contribution of each neighboring gene was calculated by summing the SHAP values for all of its features. After summing SHAP values for each gene neighbor, the gene neighbors that were defensive were identified using DefenseFinder, and the gene neighbors that were transposases or integrases were identified by searching for genes with 'transposase' or 'integrase' in their name, respectively.

HHblits searches

To search with HHblits, HMMs for query proteins were built by searching the UniRef30_2019_11 database with three iterations of HHblits. These built HMMs were then used to search the Pfam35 (*72*) database from hh-suite (*73*), or a custom-built database of known defense proteins. To build a database of HHblits-compatible HMMs of known defense proteins, the UniRef50 (*74*) dataset was searched for homologs of each known defense protein using HMMER (*75*). The sequence with the highest score was selected for each known defense protein, and these sequences were used to search the UniRef30_2019_11 database with three iterations of HHblits to build HMMs.

Structure searches

To identify structural homology between predicted defense proteins and known defense proteins, we predicted the structure of a single representative sequence for each protein cluster that was predicted defensive and all known defense proteins using ESMFold. Protein lengths were truncated at 750 amino acids due to memory constraints. The predicted structures of the predicted uncharacterized defense proteins were used to query the predicted structures of known defense proteins using Foldseek with the parameter `-alignment-type 1`. We defined homologs using a probability cutoff of 0.6 and a coverage cutoff of 0.4.

Selection of defense systems for experimental validation

TUs for each predicted defense gene were defined by taking groups of consecutive genes, transcribed in the same direction, and each separated by 30 base pairs (bps) or less. To capture the native promoter of each TU, we included 200 bp upstream of the 5'-most gene, or until the next gene was reached if it was closer. To capture the terminator of each TU, we included 100 bp downstream of the 3'-most gene, or, again, until the next gene if it was closer. Some TU boundaries were adjusted after manual inspection.

TUs were originally selected so that each TU had at least one predicted uncharacterized defense gene, however after updating DefensePredictor and improving our remote homology searches, ultimately 73, 8, and 13 selected TUs had at least one predicted uncharacterized defense gene, structural homolog, or remote homolog, respectively. 38 of these TUs were randomly selected for validation. 23 TUs were selected for having a high log-odds of defense. 16 TUs were selected for the presence of novel domains. Finally, 17 TUs were hand selected based on domain annotations. Four selected TUs were not cloned because they were found in type VI secretion system regions and had homology to known effector toxin or immunity proteins (table S6), likely representing false positives. In our calculation of validation rate these TUs were counted as unvalidated.

Selection of remote homologs for experimental validation

To select PDDEXK homologs that were predicted to be non-defensive, we identified all PDDEXK homologs in our collection of *E. coli* using HMMER. We performed our search using all Pfam HMMs belonging to the PDDEXK clan (n = 154), and used an e-value cutoff of 1E-2 to define homologs. We then randomly selected six homologs with a DefensePredictor log-odds less than 0 to experimentally test. We defined TUs for these selected homologs as above.

To identify homologs of known defense proteins more broadly, we queried one representative protein per protein cluster in our *E. coli* collection with profiles of known defense proteins using HHblits. We randomly selected 18 remote homologs with an HHblits log-odds > 0 and a DefensePredictor log-odds < 0, such that each selected homolog aligned to a different known defense protein. Two selected TUs were not cloned due to their close homology to known non-defense proteins (see table S6). One TU was clearly homologous to a TnpB-like nuclease-dead repressor (TldR) (*76*), and a second to the *tra* operon (*77*), which is responsible for facilitating the interbacterial transfer of plasmids. In our calculation of validation rate these TUs were counted as unvalidated.

Phage taxonomy construction

The phage phylogenetic tree was built using VICTOR (*78*). Phage genomes from NCBI were compared at the DNA level. Phage taxonomy was built using VICTOR's D0 algorithm, which compares phages based on the length of all homologous regions divided by genome length. Phages for screening were selected to maximize phylogenetic diversity.

Bacterial and phage culture conditions

E. coli cells were grown in Luria Broth (LB) at 37 ºC unless otherwise noted. Select ECOR strains were obtained from the Thomas S. Whittam STEC Center at Michigan State University (*22*), and UMB isolates were obtained from Alan J. Wolfe at Loyola University Chicago (*79*).

Phages were propagated overnight from single plaques. To harvest phages, cultures were treated with chloroform, spun down, and the resulting supernatant was extracted. The identity of each phage was confirmed by PCR (primers in table S11). Phages were stored at 4 ºC. Phage phiLS50-11 (referred to here as Candy) was isolated during Harvard's LS50 course in the fall of 2021 by students under the instruction of S. Srikant. It was isolated from Charles River water on *E. coli* MG1655, and sequenced to taxonomically classify it as *Tevenvirinae*.

Strain construction

TUs were PCR amplified from their native strains (table S11). Gibson assembly was used to insert defense systems into pCV1 (*8*), which has a pSC101 origin of replication, chloramphenicol resistance cassette, and no promoters upstream of the insertion site. Three remote homologs of known defense proteins were cloned into pBAD, which has an upstream arabinose-inducible promoter, because they were unlikely to function with upstream genes based on WebFlaGs analysis. Plasmids were verified by whole plasmid sequencing and transformed into *E. coli* MG1655 for screening.

Catalytic residues were identified by aligning with previously characterized proteins. Mutations to catalytic residues were generated using site-directed mutagenesis. For each catalytic mutant, completely overlapping PCR primers facing opposite directions and containing the desired

mutation were designed. Each PCR was run for 18 cycles, and the linearized product was transformed into *E. coli* DH5α cells. Re-circularized products were verified by whole plasmid sequencing and transformed into *E. coli* MG1655. The same process was used to delete plasmid regions, except outward-facing primers were designed flanking each region-to-be-deleted and each primer contained homology at their 5' end to the other side of the region.

Efficiency of plating assays

To test for anti-phage defense, strains were grown overnight in LB, diluted 1:100 into melted LB + 0.5% agar, plated onto LB + 2% agar, and left for 30 minutes at 22 ºC. Eight 10-fold serial dilutions were generated for each phage, and 2 μL of each dilution was dispensed onto *E. coli* lawns. Plates were incubated overnight at 37 ºC. Plaque forming units (PFUs) were measured by counting the number of individual plaques at the most concentrated dilution where such plaques were discernable and multiplying this count by the dilution factor (table S7). In instances where individual plaques bled into each other, the outer contours of plaques were used to count. If plaques could not be discerned at the lowest visible dilution, then a default count of 50 was used. EOP for each predicted defense system was calculated by dividing its PFU by the PFU of an empty vector strain.

TUs that did not validate in LB at 37 ºC from the set of 38 randomly selected TUs were screened in slow growth conditions, where LB medium was replaced with M9L medium (M9 salts supplemented with 0.2 mM $CaCl_2$, 2 mM $MgSO_4$, 0.1% casamino acids, and 0.4% glycerol), and cells were grown at 30 ºC. The slow growth condition yielded on new positive hit (DS-30).

Annotating validated defense systems

Domains for validated proteins were comprehensively annotated using the web tool Hhpred (*80*) to search the Pfam database, the Protein Data Bank (PDB) (*81*), the Structural Classification of Proteins (SCOPe) database (*82*), and the Conserved Domain Database (CDD) (*83*). Hhpred uses HHblits to carry out these searches, but it also incorporates information about each sequence's predicted secondary structure, making it slightly more sensitive than the local HHblits searches. The highest probability domain was selected for each non-overlapping protein region. The label for each domain was curated based on all hits to the same region. Transmembrane regions of validated proteins were identified using DeepTMHMM (*84*). Coiled-coil regions were identified using DeepCoil2 (*85*). The PD-(D/E)XK motif was identified for each validated protein using HHPred alignments to characterized nucleases. The extracted PD-(D/E)XK motifs were aligned using Kalign (*86*) and visualized using JalView (*87*).

Analyzing taxonomic distribution of validated systems

HMMs of each protein in a validated system were built by searching the UniRef30_2019_11 database with HHblits. HMMs were used to search for homologs across the ~17,000 representative genomes using HMMER. Homologs were identified using an E-value cutoff of 0.001 and a coverage cutoff of 80% for query and target sequences. For multi-gene systems, system completeness was assessed by examining whether homologs of the same system were encoded next to each other in the genomes they were found in.

Protein structure prediction and visualization

Protein structures were predicted using the AlphaFold3 server (*88*). Protein structures were visualized using ChimeraX (*89*). Structural alignments were generated using the matchmaker command in ChimeraX with default parameters. The predicted structures of the PD-(D/E)XK nucleases were aligned using the first aspartate in their catalytic motif, as most validated nucleases did not share enough homology to align using ChimeraX's matchmaker.

Predicting defense systems in set of 3,000 E. coli and 1,000 diverse prokaryotic strains

3,000 assembled *E. coli* or *Shigella* genomes were randomly selected and downloaded from NCBI. DefensePredictor was run on all genomes. Proteins were clustered at 30% sequence identity and 80% reciprocal coverage using MMseqs2. Protein clusters were categorized as in Fig. 2B, such that if any cluster member was an "expected homolog" this designation was given to the cluster, then "homolog in a new context," then "remote homolog," and finally "uncharacterized" if all predicted defensive cluster members were designated as such. The unique predicted uncharacterized defense proteins were searched for homologs using HMMER, with profiles from DefenseFinder 2.0.1 and PADLOC 2.0.0, using e-value and coverage cutoffs specified by each tool. Phage Defence Candidate (PDC) systems (*33*) were excluded from the PADLOC search. The 1,000 diverse prokaryotic strains were randomly selected from the set of ~17,000 representatives and analyzed in the same manner.

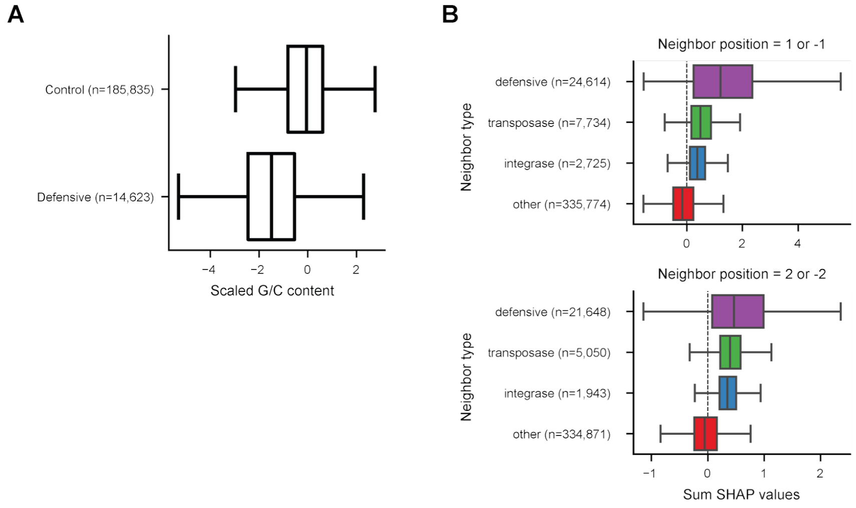

Fig. S1. DefensePredictor leverages known genomic associations to make its predictions. (**A**) Box plot of Z-scored GC content of genes across all cross-validation folds, split based on which genes are defensive. Each box represents the interquartile range, the median is shown, and whiskers represent the furthest data point within 1.5 times the interquartile range. The number of genes in each group is indicated. (**B**) Box plot of summed SHAP values for neighboring genes one or two away that are labeled as defensive, transposases, or integrases for all genes in held-out folds. Each box represents the interquartile range, the median is shown, and whiskers represent the furthest data point within 1.5 times the interquartile range. The number of genes in each category is indicated.

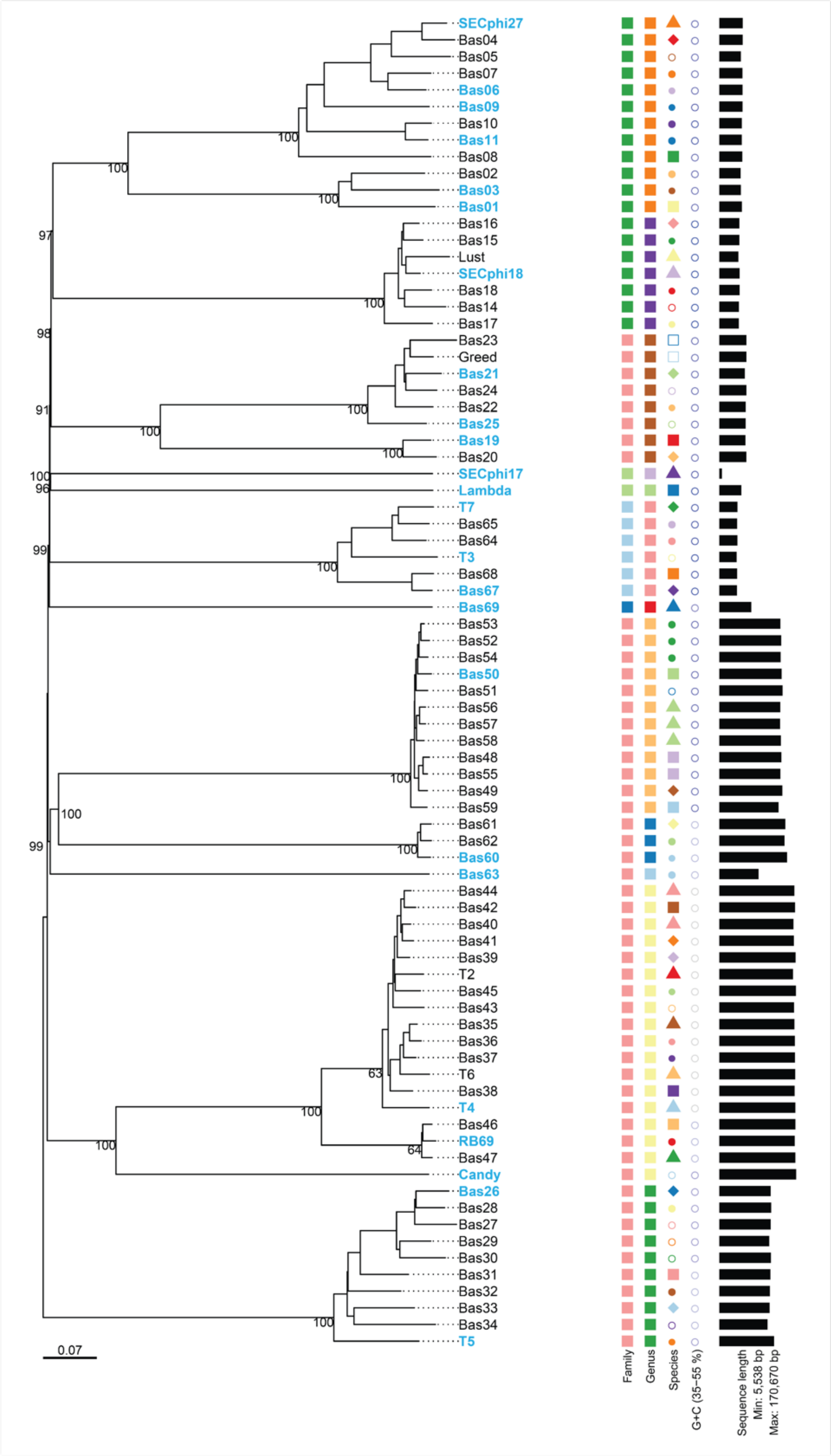

Fig. S2. Selected phages represent a broad range of taxonomies.

Phylogenetic tree of BASEL (*90*) and other common *E. coli* phage. Phages selected for screening are shown in blue. Family, genus, and species groups inferred from the tree are indicated. GC content is indicated, where low and high GC content genomes are represented by light gray and dark blue circles, respectively. Genome length is also indicated.

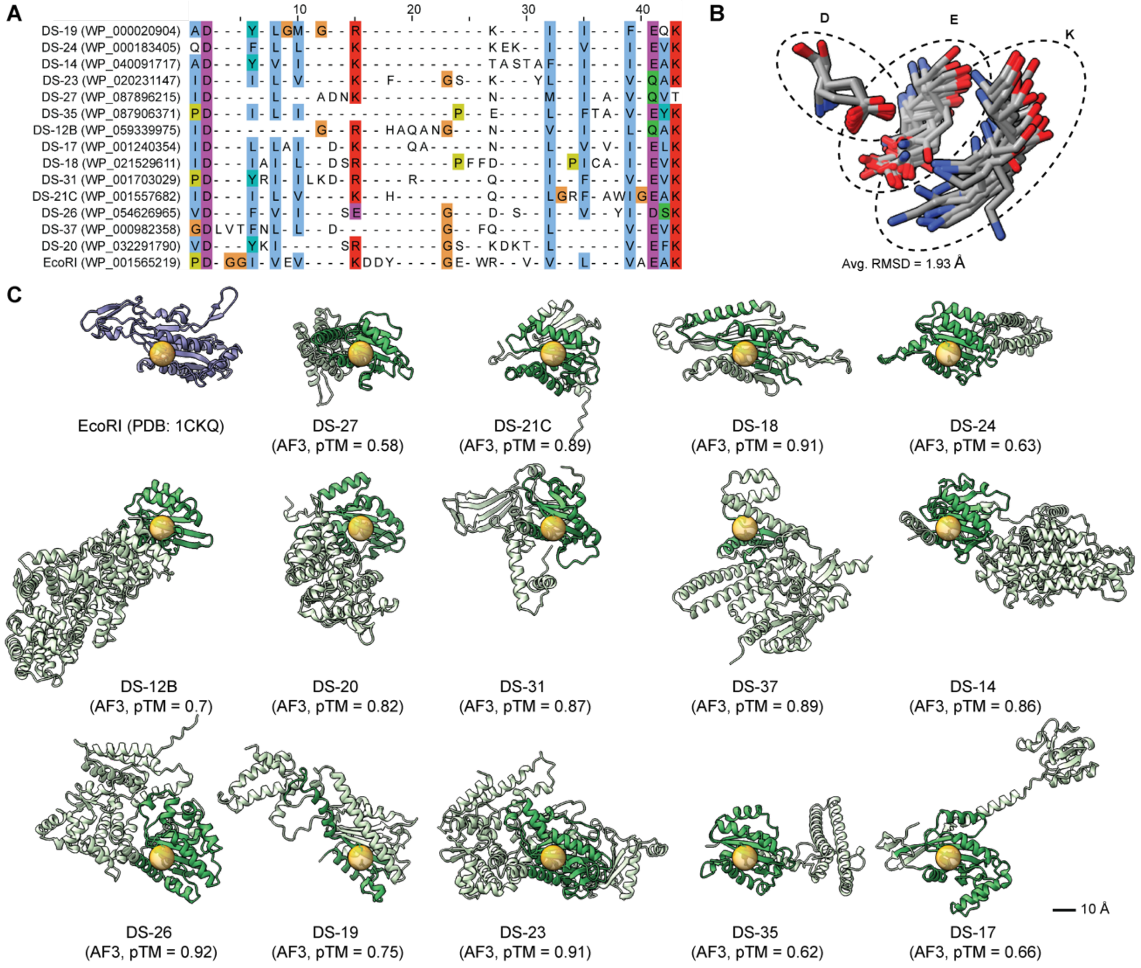

Fig. S3. Validated PD-(D/E)XK nucleases have diverse predicted structures.

(**A**) Sequence alignment of PD-(D/E)XK motifs. Protein accessions are indicated. Residues are colored based on biochemical properties using the ClustalX coloring scheme: blue – hydrophobic, red – positive charge, magenta – negative charge. (**B**) Alignment of catalytic residues from the predicted structures of the PD-(D/E)XK nucleases and the solved structure of EcoRI (PDB: 1CKQ). Aligning residues are circled and labeled with the canonical residue for that position. (**C**) Predicted structures for validated PD-(D/E)XK nucleases and the solved structure of EcoRI. The catalytic core of each protein is indicated with an orange sphere. PD-(D/E)XK domains are highlighted in green. Scale bar is shown.

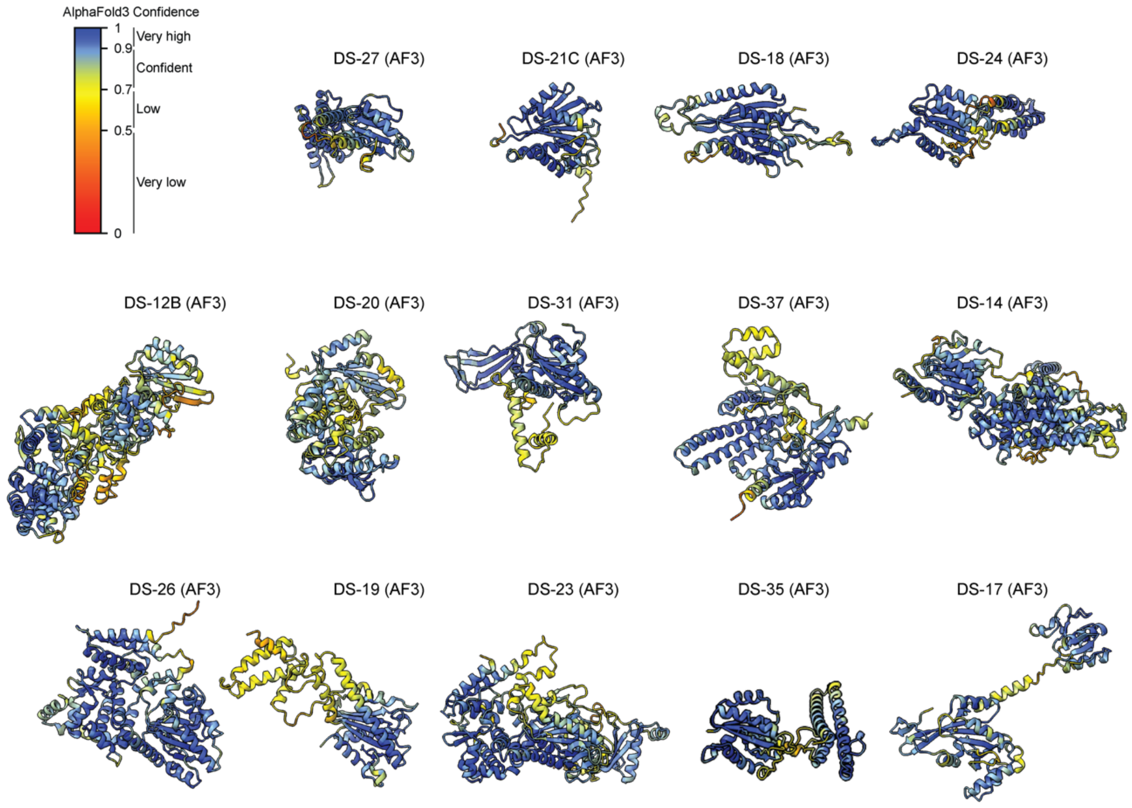

Fig. S4. AlphaFold3 confidence metrics for PD-(D/E)XK nucleases.

Per-residue pLDDT confidence scores mapped onto the predicted structures of the indicated PD-(D/E)XK nucleases.

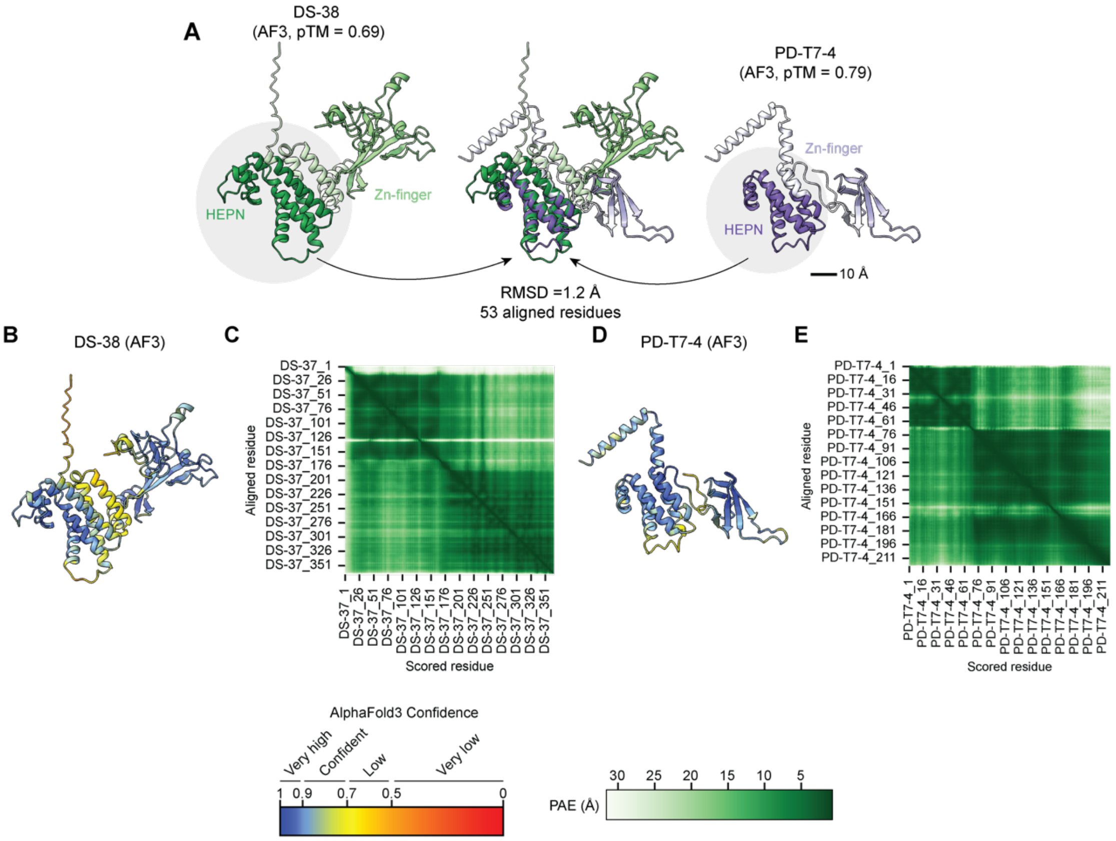

Fig. S5. DS-38 and PD-T7-4 have distinct Zn-finger domains.

(**A**) Left: predicted structure of DS-38 with domains annotated in dark green. Right: predicted structure of PD-T7-4 with domains annotated in dark blue. Center: Alignment between DS-38 and PD-T7-4. Scale bar is shown. (**B**, **D**) Per-residue pLDDT confidence scores mapped onto the predicted structures for DS-38 (**B**) and PD-T7-4 (**D**). (**C**, **E**) Predicted aligned error (PAE) between residues for DS-38 (**C**) and PD-T7-4 (**E**).

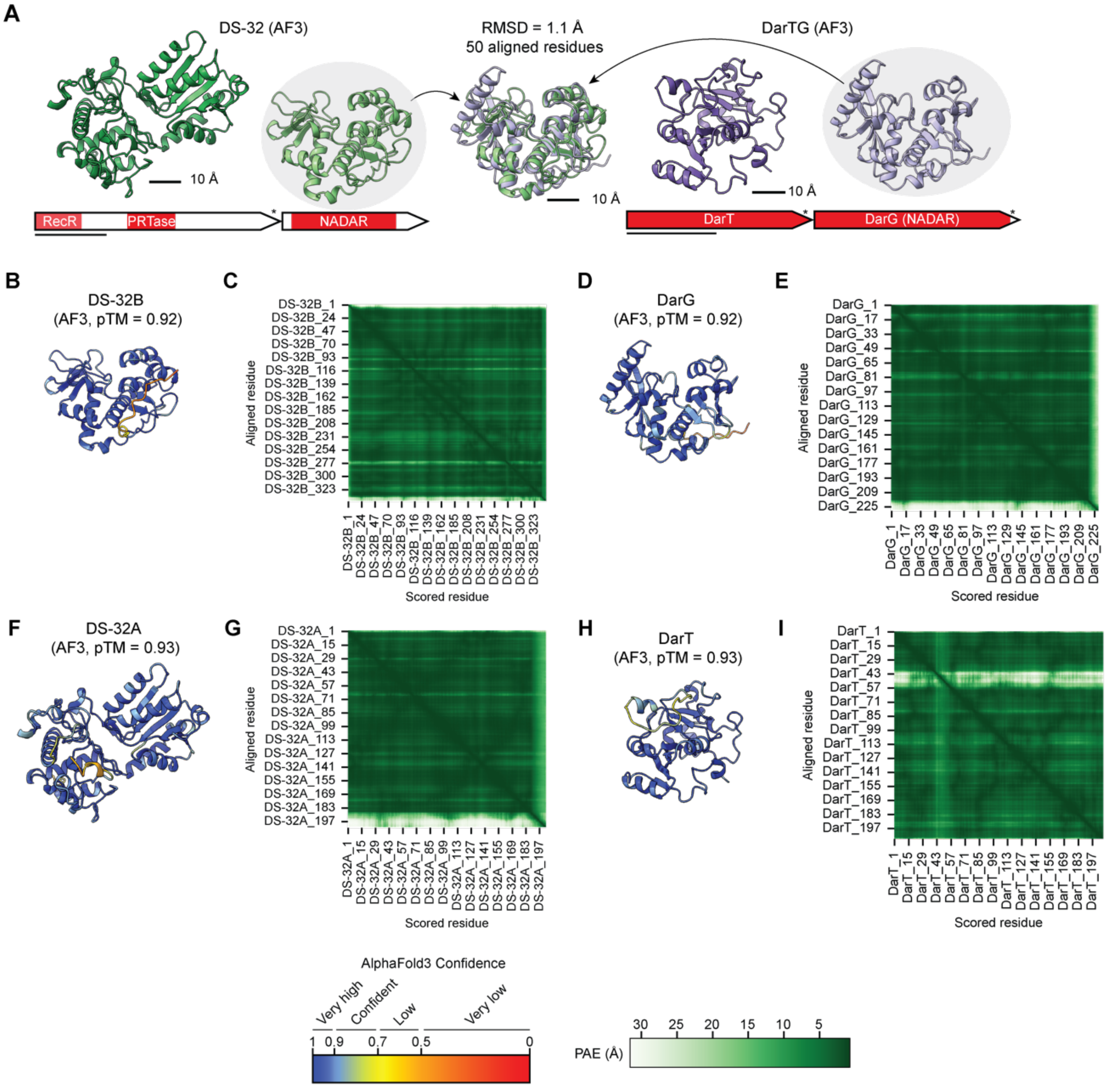

Fig. S6. DS-32 and DarTG only have homologous NADAR proteins.

(**A**) Left/bottom: domain annotations for DS-32. Left/top: predicted structures of DS-32 genes. Right: Same as left for DarTG. Center: Alignment between predicted structures of DS-32B and DarG. Scale bar is shown. (**B**, **D**, **F**, **H**) Per-residue pLDDT confidence scores mapped onto the predicted structures for DS-32B (**B**), DarG (**D**), DS-32A (**F**), and DarT (**H**). (**C**, **E**, **G**, **I**). Predicted aligned error (PAE) between residues for DS-32B (**C**), DarG (**E**), DS-32A (**G**), and DarT (**I**).

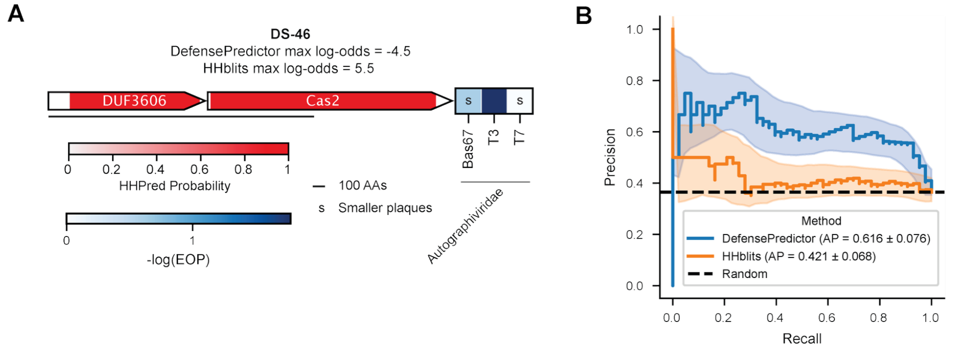

Fig. S7. DefensePredictor has better precision and recall than a remote homology approach.

(**A**) Defense heatmap (as in Fig. 3C) for DS-46. Only phages that were defended against are shown. (**B**) Precision-recall curves for DefensePredictor and HHblits evaluated on experimental screening data. True positives and negatives represent validated and unvalidated defense systems, respectively. Shaded area represents the standard deviation of precision across shared recall cutoffs for 100 bootstrap resamplings of the screening data. Average precision (AP) and the standard deviation of AP estimated from 100 bootstrap resamplings are indicated.

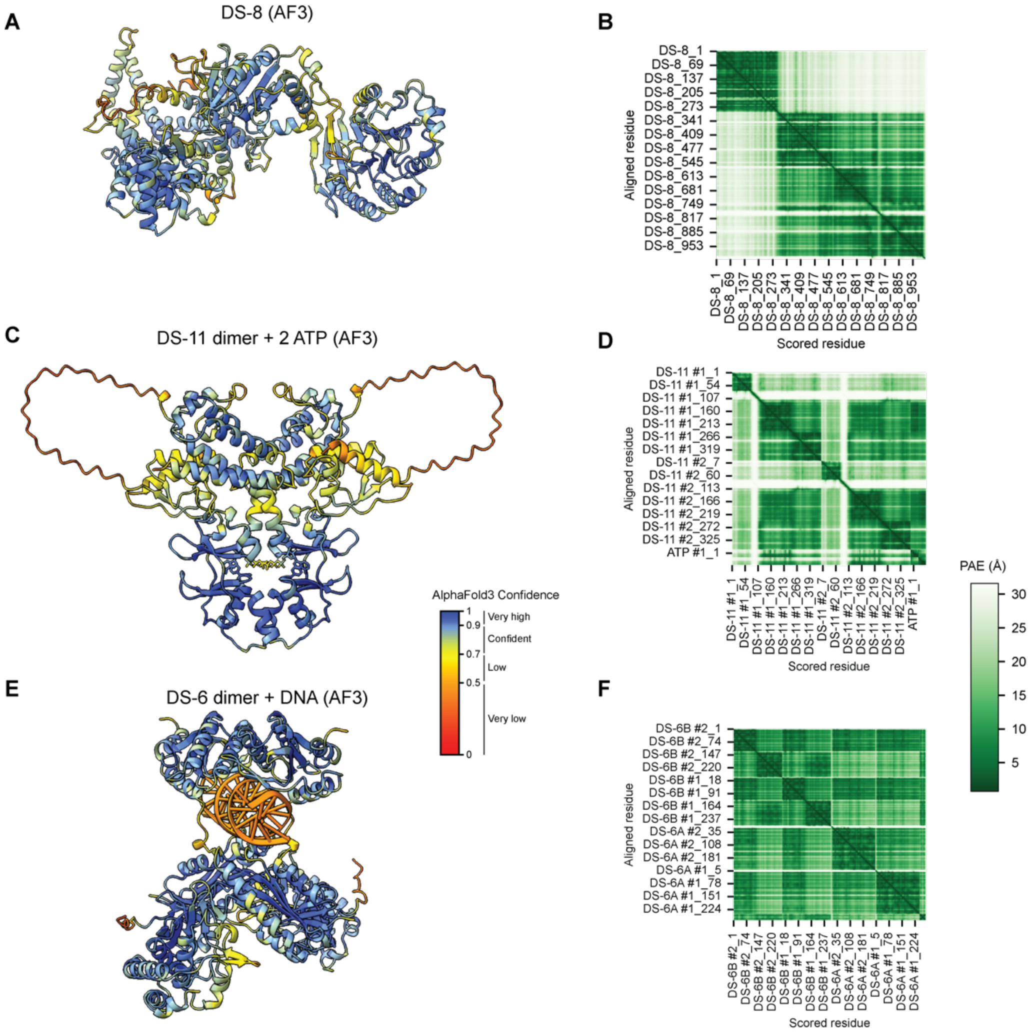

Fig. S8. AlphaFold3 confidence metrics for select systems with novel defense domains. (**A**, **C**, **E**) Per-residue pLDDT confidence scores mapped onto the predicted structures for DS-8 (**A**), two copies each of DS-11 and ATP (**C**), two copies of DS-6 and DNA (**E**). (**B**, **D**, **F**, **H**) Predicted aligned error (PAE) between residues for DS-8 (**B**), two copies each of DS-11 and ATP (**D**), two copies of DS-6 and DNA (**F**).

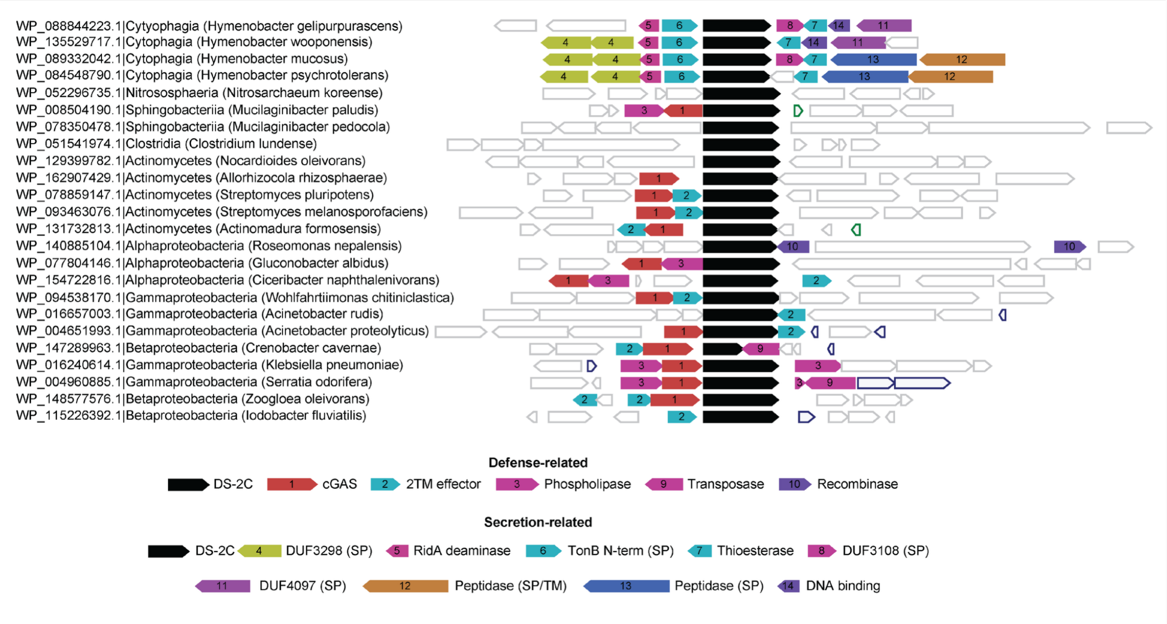

Fig. S9. A di-adenylate cyclase co-occurs with CBASS genes.

WebFlaGs output showing homologs of *DS-2C* and their genomic neighbors. The protein accession and species for each homolog is indicated. Clusters of neighboring genes share the same number and are labeled at the bottom of the plot. SP, signal peptide-containing protein; TM, transmembrane domain-containing protein.

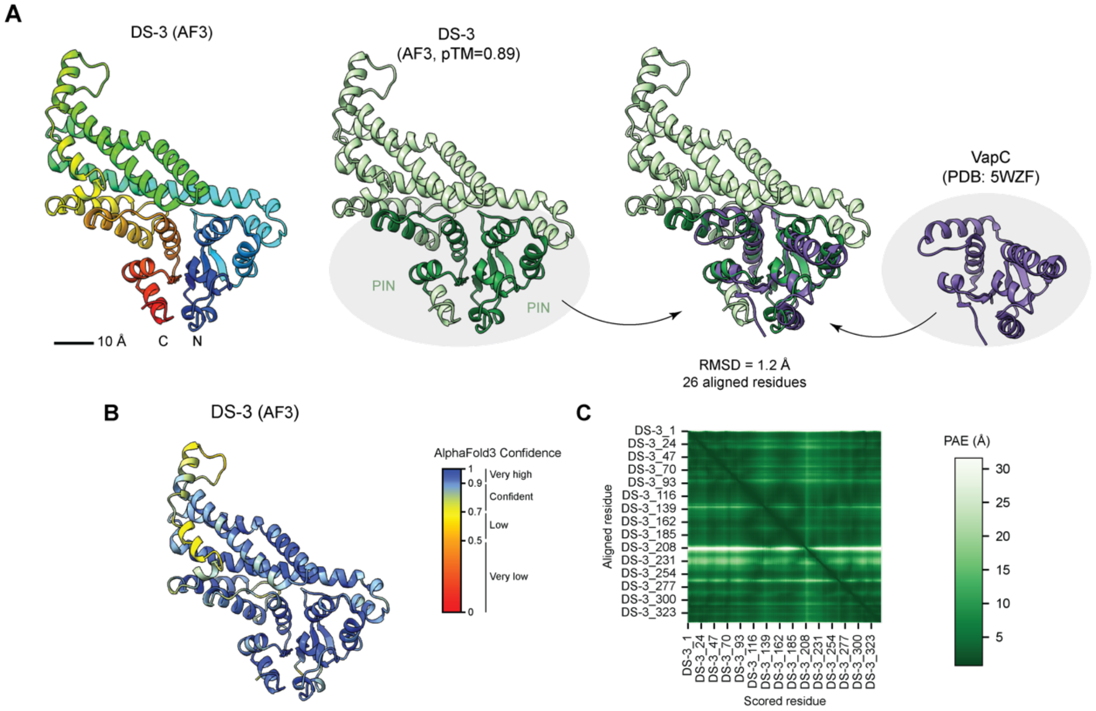

Figure S10. DS-3 has a PIN domain split between its N and C-terminus.

(**A**) Predicted structure of DS-3, colored blue to red to indicate its N versus C-terminus, respectively (left), or with both halves of its PIN domain colored green (center). The predicted structure of DS-3 is compared with the solved structure of VapC from *Mycobacterium tuberculosis* (*91*). Scale bar is shown. (**B**) Per-residue pLDDT confidence scores mapped onto the predicted structure of DS-3 (**C**) Predicted aligned error (PAE) between DS-3 residues.

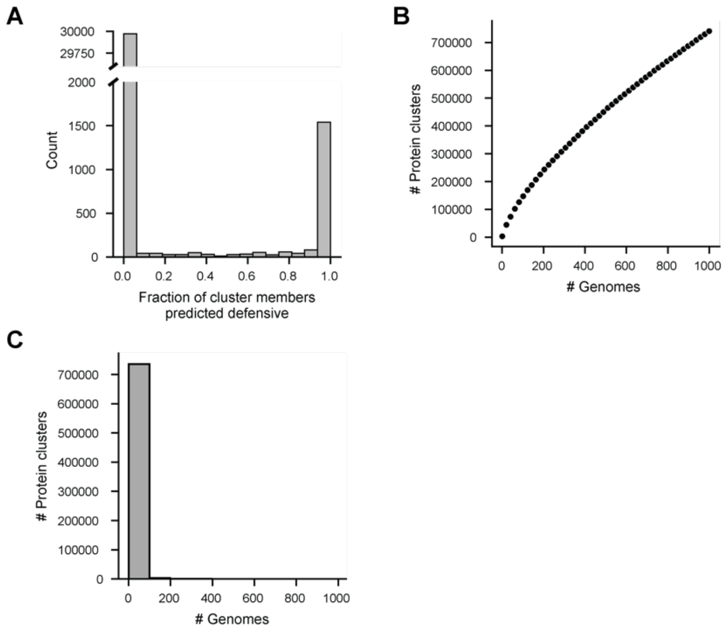

Figure S11. DefensePredictor outputs similar predictions for homologs.

(**A**) Histogram of the fraction of protein cluster members predicted as defensive. Only clusters with three or more members from a set of 3,000 *E. coli* strains were considered. (**B**) Average number of protein clusters when considering between 1 and 1,000 diverse prokaryotic genomes in steps of 50. Averages were calculated by resampling genomes 10 times at each step. (**C**) Histogram of the number of genomes each protein cluster is encoded by in a set of 1,000 diverse prokaryotic strains.

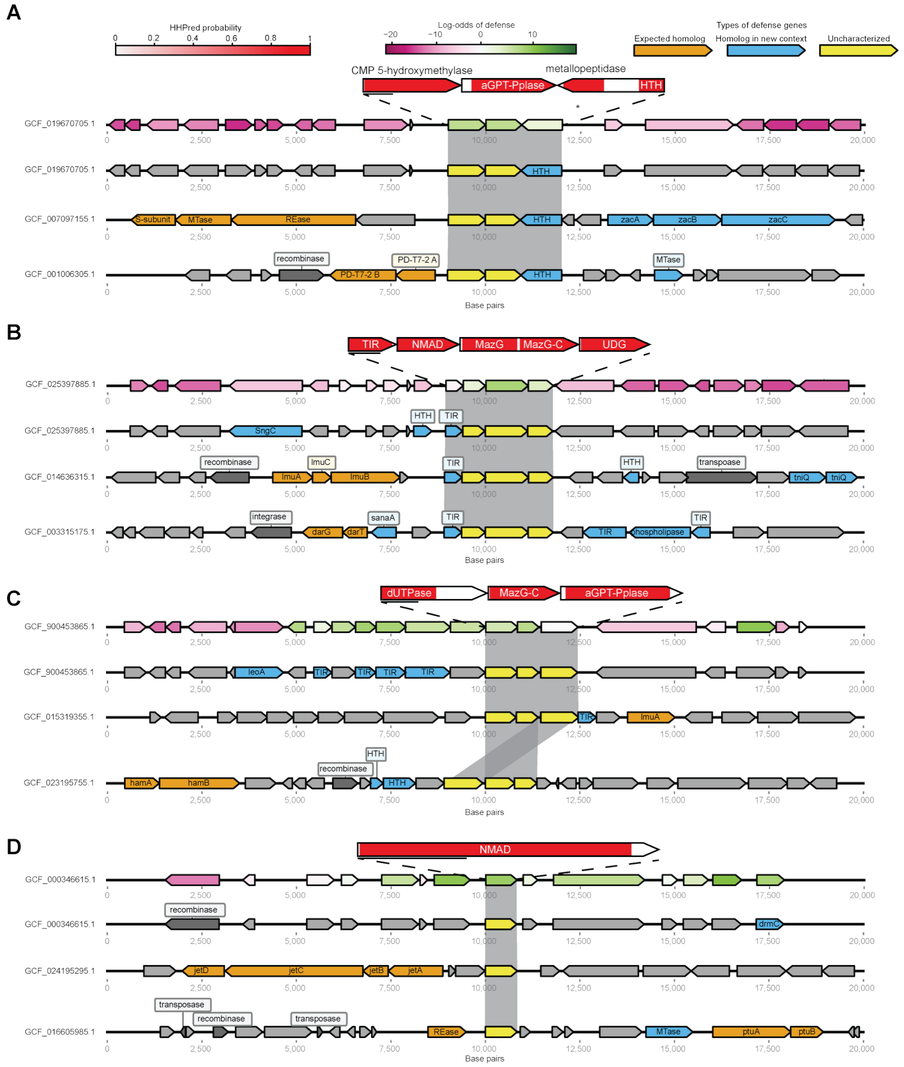

Figure S12. Predicted defense systems co-occur with known systems.

(**A**) Top: domain annotations for a predicted defense system with an alpha-putrescinyl/glutamylthymidine pyrophosphorylase (aGPT-Pplase) domain. Horizontal scale bar represents 100 amino acids. Gene with an asterisk represents a homolog of a known defense gene. First genomic region: DefensePredictor log-odds for the genomic region in the indicated genome. Green genes are predicted defensive. Genes delineated by dashed lines represent the predicted system. Second genomic region: same as the first genomic region, but genes are colored based on homology to known defense genes. Final two genomic regions: additional genomic regions where homologs of the highlighted system are found. Shaded areas between genomic regions represent regions of homology. aGPT-Pplase, alpha-putrescinyl/glutamylthymidine pyrophosphorylase. (**B**) Same as (**A**) for a predicted system with a NTP pyrophosphoydrolase (MazG) and a predicted kinase domain (MazG-C). NMAD, nucleotide modification associated domain; UDG, uracil DNA gycosylase. (**C**) Same as (**A**) for a predicted system with a predicted kinase (MazG-C), dUTPase, and aGPT-Pplase domain. (**D**) Same as (**A**) for a predicted system with a nucleotide modification associated domain (NMAD).

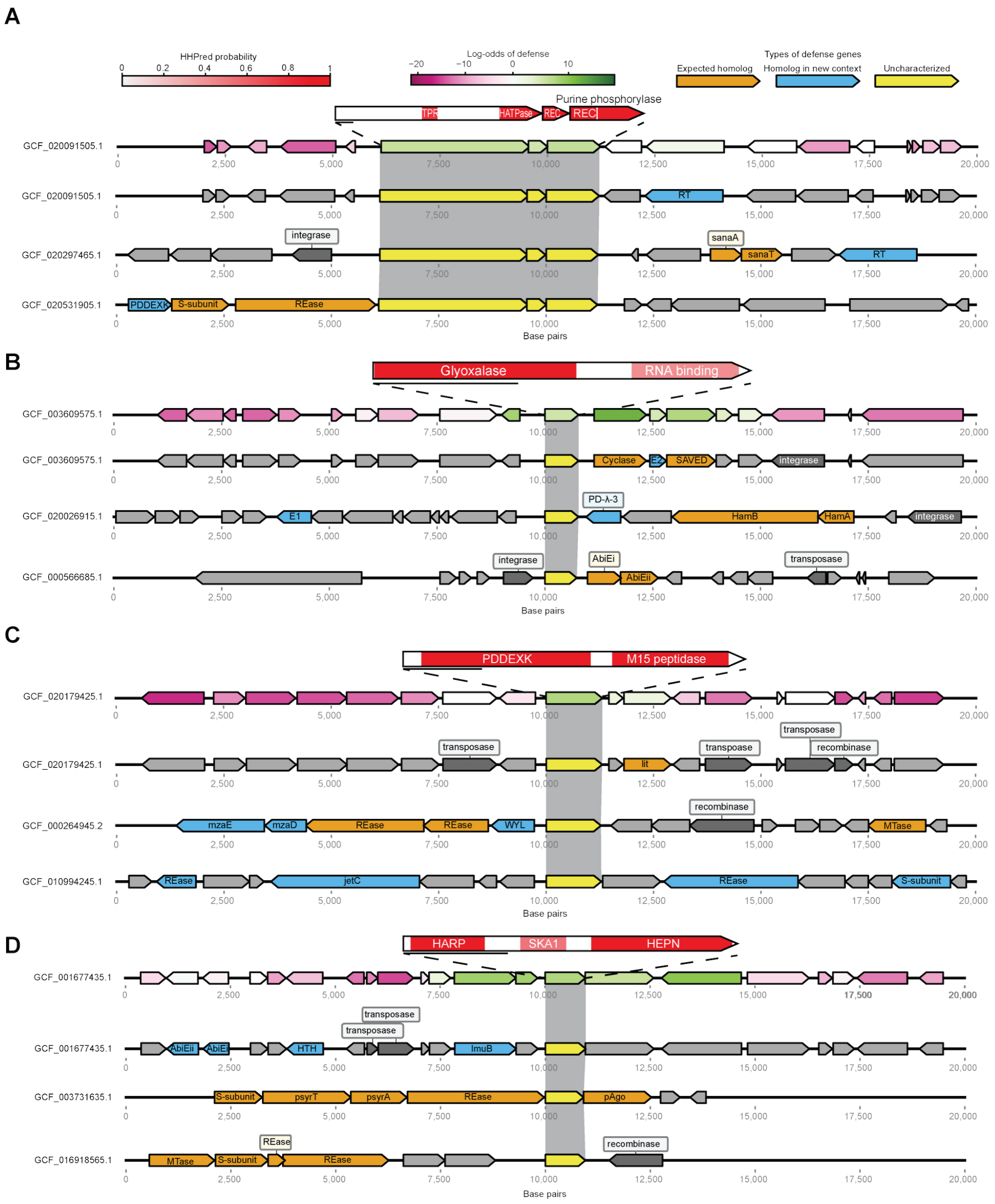

Figure S13. Predicted defense systems co-occur with known systems.
(**A**) Same as fig. S12A, but for a predicted defense system with a purine phosphorylase domain. TPR, tetratricopeptide repeat; HATPase, histidine kinase-like ATPase; REC, receiver domain. (**B**) Same as (**A**) for a predicted system with a glyoxalase domain. (**C**) Same as (**A**) for a predicted system with a M15 peptidase domain. (**D**) Same as (**A**) for a predicted system with a HARP domain. SKA1, spindle and kinetochore associated protein.

Titles of Tables S1-S11

Table S1. Dataset for model development.

Table S2. Model performance metrics.

Table S3. SHAP values.

Table S4. Predictions for 1,000 diverse prokaryotic genomes.

Table S5. Predictions for 69 diverse *E. coli* strains.

Table S6. TUs selected for screening.

Table S7. Plaquing assay quantification.

Table S8. Protein domains in validated systems.

Table S9. Summary statistics from AlphaFold3 predictions.

Table S10. Predictions for 3,000 *E. coli* strains.

Table S11. Primer sequences and created strains.
